# Defining the mutation signatures of DNA polymerase θ in cancer genomes

**DOI:** 10.1093/narcan/zcaa017

**Published:** 2020-08-27

**Authors:** Taejoo Hwang, Shelley Reh, Yerkin Dunbayev, Yi Zhong, Yoko Takata, Jianjun Shen, Kevin M McBride, John P Murnane, Jong Bhak, Semin Lee, Richard D Wood, Kei-ichi Takata

**Affiliations:** School of Life Sciences, Ulsan National Institute of Science and Technology, Ulsan 44919, Republic of Korea; Department of Epigenetics & Molecular Carcinogenesis, The University of Texas MD Anderson Cancer Center, Smithville, TX 78957, USA; School of Life Sciences, Ulsan National Institute of Science and Technology, Ulsan 44919, Republic of Korea; Center for Genomic Integrity, Institute for Basic Science, Ulsan 44919, Republic of Korea; Department of Epigenetics & Molecular Carcinogenesis, The University of Texas MD Anderson Cancer Center, Smithville, TX 78957, USA; Department of Epigenetics & Molecular Carcinogenesis, The University of Texas MD Anderson Cancer Center, Smithville, TX 78957, USA; Department of Epigenetics & Molecular Carcinogenesis, The University of Texas MD Anderson Cancer Center, Smithville, TX 78957, USA; Department of Epigenetics & Molecular Carcinogenesis, The University of Texas MD Anderson Cancer Center, Smithville, TX 78957, USA; Department of Radiation Oncology, University of California, San Francisco, San Francisco, CA 94143, USA; School of Life Sciences, Ulsan National Institute of Science and Technology, Ulsan 44919, Republic of Korea; Korean Genomics Center, Ulsan National Institute of Science and Technology, Ulsan 44919, Republic of Korea; Personal Genomics Institute, Genome Research Foundation, Cheongju 28160, Republic of Korea; Clinomics Ltd, Ulsan National Institute of Science and Technology, Ulsan 44919, Republic of Korea; School of Life Sciences, Ulsan National Institute of Science and Technology, Ulsan 44919, Republic of Korea; Korean Genomics Center, Ulsan National Institute of Science and Technology, Ulsan 44919, Republic of Korea; Department of Epigenetics & Molecular Carcinogenesis, The University of Texas MD Anderson Cancer Center, Smithville, TX 78957, USA; School of Life Sciences, Ulsan National Institute of Science and Technology, Ulsan 44919, Republic of Korea; Center for Genomic Integrity, Institute for Basic Science, Ulsan 44919, Republic of Korea

## Abstract

DNA polymerase theta (POLQ)-mediated end joining (TMEJ) is a distinct pathway for mediating DNA double-strand break (DSB) repair. TMEJ is required for the viability of *BRCA*-mutated cancer cells. It is crucial to identify tumors that rely on POLQ activity for DSB repair, because such tumors are defective in other DSB repair pathways and have predicted sensitivity to POLQ inhibition and to cancer therapies that produce DSBs. We define here the *POLQ*-associated mutation signatures in human cancers, characterized by short insertions and deletions in a specific range of microhomologies. By analyzing 82 COSMIC (Catalogue of Somatic Mutations in Cancer) signatures, we found that *BRCA*-mutated cancers with a higher level of *POLQ* expression have a greatly enhanced representation of the small insertion and deletion signature 6, as well as single base substitution signature 3. Using human cancer cells with disruptions of *POLQ*, we further show that TMEJ dominates end joining of two separated DSBs (distal EJ). Templated insertions with microhomology are enriched in POLQ-dependent distal EJ. The use of this signature analysis will aid in identifying tumors relying on POLQ activity.

## INTRODUCTION

**Figure 1.**
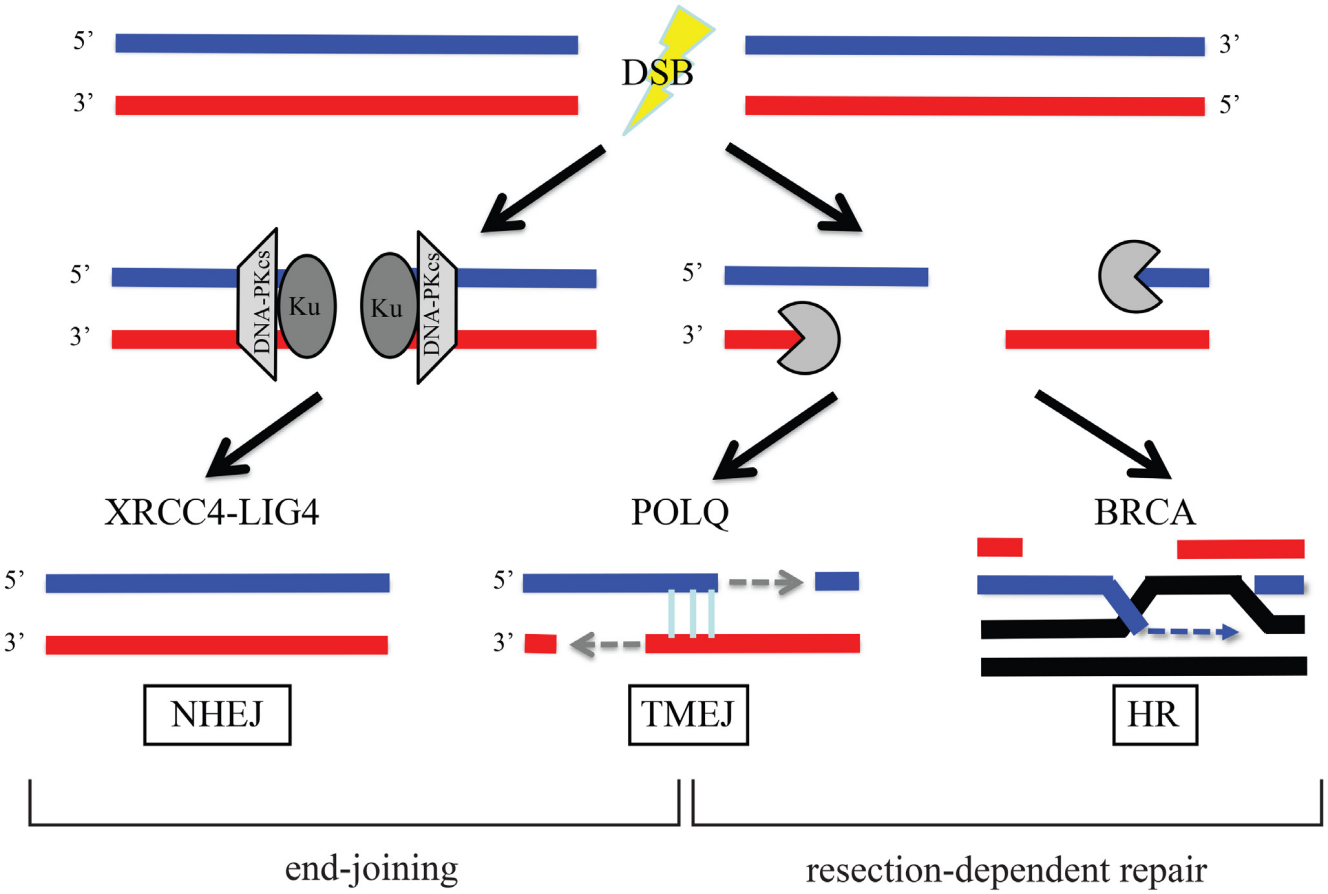
POLQ is an important alternative for NHEJ or HR. Upon DSB formation, NHEJ factors inhibit DNA resection and stimulate end joining. When DSB damage is unable to be repaired by NHEJ, 3′ DNA tails are generated from DNA end resection by nucleases. The 3′ DNA tails are processed by TMEJ or BRCA-mediated HR. POLQ becomes more important when NHEJ or HR is not available and repairs DSBs at the expense of introducing mutation signatures. Detection of those signatures in tumor biopsy may be a useful approach to influence treatment decision.

DNA double-strand breaks (DSBs) are deleterious lesions that can lead to cell death if not repaired. Additionally, DSB repair processes have the potential to introduce mutations and chromosome rearrangements. There are three major pathways for repair of DSBs: (i) nonhomologous end joining (NHEJ), (ii) homologous recombination (HR) and (iii) DNA polymerase theta (θ)-mediated end joining (TMEJ; Figure 1). NHEJ is often the predominant pathway for the repair of DSBs that occur outside of replication (1,2). In the absence of NHEJ, the broken ends are resected by nucleases and then the exposed single-stranded DNA (ssDNA) tails are processed by HR or TMEJ. HR uses an undamaged homologous DNA template to repair DNA initiated from ssDNA tails, as does the related single-strand annealing (SSA) process (1,3). TMEJ mediates the joining of two resected 3′ ends harboring DNA sequence microhomology (MH) in a Ku-independent manner (4). NHEJ and TMEJ can occur throughout the cell cycle but HR operates only in S and G_2_ stages when a sister chromatid is present (5,6). The choice of DSB repair pathway influences the fidelity of DSB repair, which eventually influences the rate of tumorigenesis. TMEJ is an important alternative to the major DSB repair pathways, HR and NHEJ. The requirement of POLQ for the viability of *BRCA*-mutated cancer cells underscores the importance of TMEJ (7). Accordingly, tumors with disrupted HR or NHEJ pathways, including BRCA1/2, rely on POLQ activity for DSB repair, and are sensitive to POLQ inhibition, PARP inhibition or cancer therapies introducing DSBs (7–9). To guide personalized therapies, there is an urgent need to have a general method to identify tumors that rely on POLQ activity.

POLQ is a unique multifunctional enzyme. It has an N-terminal helicase-like domain (HLD) linked to a C-terminal A-family DNA polymerase domain via a central region (10). The POLQ-HLD and polymerase domains both contribute to end joining (11–13). Although the majority of A-family DNA polymerases including *Escherichia coli* pol I and DNA polymerase γ are high-fidelity polymerases, POLQ is error-prone (14,15). POLQ harbors an exonuclease-like domain but it lacks a 3′→5′ proofreading activity (10). Because of its low fidelity and the unique thumb domain that carries positively charged residues to grasp the unstable primer terminus, POLQ can extend DNA from mismatched primers (16).

DNA repair, including TMEJ, is important to avoid cell death from DNA damage. DNA repair processes are not always perfect, however, and can contribute to base substitutions, deletions and insertions in cancer genomes. Sequencing technologies have helped to decipher patterns of somatic mutations in cancer genomes (17,18). Originally, 30 independent COSMIC (Catalogue of Somatic Mutations in Cancer) signatures were identified (17). These were recently updated, and new COSMIC mutation signatures including single base substitution (SBS) and small insertion and deletion (ID) signatures are being used to characterize cancer genomes (18). SBS signatures are used to classify single-nucleotide mutations according to 96 types: 6 types of substitution (C>A, C>G, C>T, T>A, T>C, T>G) multiplied by 4 possible 5′ flanking bases (A, C, G or T) and 4 possible 3′ flanking bases (A, C, G or T). ID signatures are used to classify insertion and deletion mutations into 83 ID types: 12 types of 1 bp deletions, 12 types of 1 bp insertions, 24 types of ≥2 bp deletions, 24 types of ≥2 bp insertions and 11 types of ≥2 bp deletions at MHs (18). The mechanism of origin of some of these signatures is understood, but most arise by unknown mechanisms.

In TMEJ, POLQ joins broken ends utilizing MH exposed after DNA resection. This activity introduces short DNA deletions, while protecting against large catastrophic deletions (11,19). This unique activity of POLQ yields a distinctive mutation pattern that has been proposed to be related to COSMIC3, a mutational signature often found in *BRCA*-mutated cancers (20,21). This mutation signature is associated with up to 50 bp deletions with overlapping MHs at end-joining sites (17,18). *POLQ* mRNA overexpression has been reported in human malignancies (22,23). However, a clear demonstration that *POLQ* status influences the occurrence of the COSMIC3 signature in tumors is currently lacking.

Here, we describe experiments to analyze which COSMIC signatures (18) are enriched among 82 signatures in *BRCA*-mutated cancers that express high levels of wild-type *POLQ*. We identified three such signatures: single base substitution signature 3 (SBS3), a recently updated version related to COSMIC3, and small insertion and deletion signatures 6 and 8 (ID6 and ID8). The detection of those signatures in whole genome data from individual tumors may be useful to monitor POLQ activity for cancer treatment. However, SBS3, ID6 and ID8 are not designed to monitor POLQ activity, so they do not comprehensively reflect POLQ mutagenesis. It is, therefore, important to improve the pipeline to define POLQ-dependent mutation signatures by more intensive analysis of the mechanism of POLQ mutagenesis.

As part of a direct exploration of the mechanism of POLQ action and mutagenesis, we identify here a situation in which POLQ is used in cells. Templated insertion is one of the hallmarks of POLQ-associated mutation (24). It is evolutionally conserved in *Drosophila* (25), *Caenorhabditis elegans* (26), mouse (27) and human (11). We show that POLQ generates templated insertions during nuclease-induced distal end joining (distal EJ), a process that promotes ligation between two separated DSBs. A physiological example of distal EJ is class switch recombination (CSR), involving two DSBs in antibody genes. During CSR in mouse B cells, POLQ introduces templated insertions of between 2 and 35 bp (27). Homologous sequences for those insertions arose from sequences directly adjacent to the resected ends or from sequences many kb distant (27). In this study, we find that insertions initiated and ended with MH are enriched in POLQ-dependent distal EJ. Distal EJ occurs when the two originally separated DSBs are not properly repaired at each DSB site by NHEJ (proximal EJ) or HR. We conclude that TMEJ is a major pathway to mediate distal EJ.

## MATERIALS AND METHODS

### Analysis of cancer mutational signatures

We used somatic single-nucleotide variant, insertion/deletion and gene expression information to distinguish samples with mutant and wild-type *POLQ*, *BRCA1* and *BRCA2*. Among the variant classifications, frameshift deletion, frameshift insertion and nonsense mutation were selected and used as somatic mutations. In the sample, if a single-nucleotide variant or an insert/deletion in a specific gene was a somatic mutation, it was called a mutant. If there was no mutation in the gene or if the mutation was not a somatic mutation, it was called a wild type. A gene expression level below the 33rd percentile was considered low and above the 33rd percentile was considered high. Somatic mutation and insertion/deletion data were obtained from the Pan-Cancer Analysis of Whole Genomes (PCAWG) consensus callsets downloaded from the ICGC Data Portal (https://dcc.icgc.org/releases/PCAWG/consensus_snv_indel). Gene expression data were from PCAWG transcriptome analysis results downloaded from the ICGC Data Portal (https://dcc.icgc.org/releases/PCAWG/transcriptome/gene_expression). Version 2 and 3 COSMIC mutational signatures were downloaded from COSMIC (https://cancer.sanger.ac.uk/cosmic/signatures). We matched the sample ID of the mutational signature data with the PCAWG consensus callset and transcriptome analysis results’ data to validate the change in signature proportion under the *POLQ*, *BRCA1* and *BRCA2* mutation or expression conditions of cancer samples. We used the Wilcoxon signed-rank test to determine which signatures are associated with *POLQ* status. We further adjusted *P*-value by the Bonferroni method for stringent statistical significance.

### CRISPR/Cas9-mediated gene disruption

We used the GeneArt CRISPR Nuclease Vector with OFP Reporter Kit from Thermo Fisher. Two oligonucleotides (5′-GATTCGTTCTCGGGAAGCGGGTTTT and 5′-CCGCTTCCCGAGAACGAATCCGGTG) that code for target-specific crRNA were annealed and ligated into the linearized GeneArt CRISPR Nuclease Vector to target exon 1 of *POLQ* (28). The GeneArt CRISPR Nuclease Vector encoding a custom single-guide RNA containing the *POLQ* targeting sequence (crRNA) and a Cas9 nuclease-recruiting sequence (tracrRNA), Cas9 nuclease and orange fluorescent protein (OFP) as a co-expression marker was transfected to DR-U2OS (29) or EDS-7F2 (30) cells with Lipofectamine LTX (Invitrogen). After the transfection, OFP-positive single cell clones were sorted into 96-well plates by flow cytometry. Genomic DNA isolated from individual clones was amplified by PCR with *POLQ* ex1 F (5′-GGGAGGTTTGAGTTTGAAGAC) and R (5′-GTCACAGAGAAGGGGAGTAG) primers. The targeted genomic DNA sequence of the complete *POLQ* knockout cell lines was confirmed by direct sequencing of PCR products amplified with *POLQ* ex1 F and R primers, and sequencing after TA cloning of the PCR products. The absence of *POLQ* in complete *POLQ* knockout cell lines was also confirmed by immunoblotting with the POLQ-specific 1B1 antibody (28,31).

### shRNA vectors

We used shRNA vectors obtained from the MD Anderson core facility: sh53BP1 (V3LHS_635694) (32), shPRKDC (DNA-PKcs) (V2LHS_94774) (28) and shControl (RHS4346).

### Antibodies

We used the following antibodies: 1B1, monoclonal anti-POLQ 1:10 000 (31); A300-516A, polyclonal anti-DNA-PKcs 1:5000; A300-272A, polyclonal anti-53BP1 1:10 000; F7425, monoclonal anti-α-Tubulin 1:8000; sc73614, monoclonal anti-vinculin 1:1000; T5168, HRP (horseradish peroxidase)-conjugated anti-mouse IgG 1:10 000; and A0168, HRP-conjugated anti-rabbit IgG 1:10 000.

### DR-GFP assays

We followed a previously published method (33). A total of 0.8 μg of the I-SceI expression vector pCBASce was transfected to 2 × 10^5^ DR-U2OS cells with Lipofectamine 2000. To determine the amount of HR, the percentage of GFP-positive cells was quantitated by flow cytometric analysis 3 days after transfection using a BD FACSDiva.

### Measuring the frequency of imprecise NHEJ of an I-SceI-generated DSB

PCR amplification for direct-repeat GFP (DR-GFP) I-SceI region was performed using pooled genomic DNA from pCBASce‐transfected cells, KOD Xtreme Hot Start DNA Polymerase (Toyobo) and the following primers: [DRGFP F] 5′-CTGCTAACCATGTTCATGCC-3′ and [DRGFP R] 5′-AAGTCGTGCTGCTTCATGTG-3′. The PCR products were incubated with I-SceI, BcgI or I-SceI + BcgI (double digestion). Cells that repair an I-SceI-generated DSB by imprecise NHEJ, SSA or HR lose the I‐SceI site. In addition, cells that repair the DSB by SSA or HR replace the I‐SceI site with a BcgI site, allowing for the discrimination of specific repair pathways (33).

### Generation of I-SceI-induced DSBs in EDS-7F2

Packaging of the pQCXIH-I-SceI retroviral vectors and infection of cell cultures were performed as previously described (30). The selection for cells infected with pQCXIH-I-SceI was achieved by growth in medium containing 50 μg/ml hygromycin (Sigma) for 14 days with medium changes every 2 days to allow for expression of I-SceI endonuclease and the generation of DSBs. The analysis of the frequency of GFP-positive (GFP+) and DsRed positive (DsRed+) cells was performed by fluorescence-activated cell sorting (FACS) using a BD FACSAria Fusion instrument (BD Biosciences). The cells were trypsinized, an equal volume of growth medium was added and they were counted and pelleted. To prevent aggregation, the cells were then resuspended in 10 ml of ice-cold Dulbecco’s phosphate-buffered saline (PBS; w/o Ca or Mg) containing 100 μg/ml Proteinase K (Sigma) by vigorous pipetting. The cells were incubated for 10 min on ice, pipetting twice more during the incubation. This treatment with Proteinase K is necessary to prevent cell aggregation. Following the incubation, 2 ml of Dulbecco’s PBS (w/o Ca or Mg) containing 1% bovine serum albumin (Sigma) was added to block further digestion with Proteinase K. The cells were then pelleted and resuspended in Dulbecco’s PBS (w/o Ca or Mg) at ∼1 × 10^6^ cells/ml for analysis by flow cytometry as reported (30).

### Sample preparation for proximal end joining

We used the GeneArt CRISPR Nuclease Vector with OFP Reporter Kit from Thermo Fisher. Two oligos (5′-CTTGCGACCTTGACCATCTTGTTTT and 5′-AAGATGGTCAAGGTCGCAAGCGGTG) were annealed and ligated to the linearized GeneArt CRISPR Nuclease Vector to target exon 6 of *HPRT*. The GeneArt CRISPR Nuclease Vector was then transfected to DR-U2OS cells with Lipofectamine LTX (Invitrogen). Forty-eight hours after the transfection, OFP (co-expression marker)-positive cells were isolated by flow cytometry. Genomic DNA was isolated from OFP-positive cells from three independent experiments and was amplified by PCR using primers (HPRTF: 5′-TCTTACTGCTTGCTGAGGGC and HPRTR: 5′-TAATTTTGCAAGGGGGCCCA) and KOD Xtreme Hot Start DNA Polymerase (Toyobo) (95°C for 2 min, followed by 35 cycles of 95°C for 30 s, 63°C for 30 s and 68°C for 45 s, last strand elongation at 68°C for 5 min). A total of 20.4 ng of genomic DNA corresponding to 3000 cells (6000 *HPRT* loci) was used for each PCR reaction, unless otherwise indicated. The formula (6.81 × 10^−12^ g/cell) determined the cell number from genomic DNA amount. The PCR products (546 bp for wild-type *HRPT* locus) were separated by 1% agarose gel and the products (100–650 bp) were cut out from the gel and purified with QIAquick gel purification kit (Qiagen).

### Sample preparation for joining at distal joined junctions in EDS-7F2 cells

After I-SceI expression, genomic DNA was isolated from GFP-positive EDS-7F2 cells. PCR amplification of the junction region was performed using pooled genomic DNA from GFP-positive EDS-7F2 cells (54 ng of genomic DNA, corresponding to 7930 cells), KOD Xtreme Hot Start DNA Polymerase (Toyobo) and the following primers: [7F2GFP NGS F] 5′-GTCCCAAATCTGGCGGAG-3′ and [7F2GFP NGS R] 5′-GTAGCGGCTGAAGCACTG-3′ (94°C for 2 min, followed by 10 cycles of 94°C for 30 s, 58°C for 30 s and 68°C for 45 s, last strand elongation at 65°C for 5 min). We considered that a single cell carries 6.81 × 10^−12^ g of genomic DNA. The 597 bp amplicons were then used for nested PCR with the following primers: [7F2GFP NGS F2] 5′-AGGAAGGAAATGGGCGGGGA-3′ and [7F2GFP NGS R2] 5′-AACTTCAGGGTCAGCTTGCC-3′ (94°C for 2 min, followed by 23 cycles of 94°C for 30 s, 62°C for 30 s and 68°C for 45 s, last strand elongation at 65°C for 5 min). The 429 bp PCR amplicons were purified by PCR purification kit (Qiagen) and eluted with 60 μl of EB buffer (10 mM Tris–Cl, pH 8.5).

### Library preparation and sequencing

Illumina sequencer compatible libraries were prepared using a Kapa Hyper Prep Kit (Roche Sequencing and Life Science) according to the manufacturers’ protocol. Briefly, 40 ng of each PCR product was end repaired and 3′-adenylated, and then ligated to NexTflex adaptors (PerkinElmer, Inc., Bioo Scientific). The constructed libraries were subjected to 0.8× AMPure XP bead clean-up and additional 0.7× AMPure XP bead clean-up (Beckman Coulter). The library quality was validated on a 2200 TapeStation from Agilent Technologies (Agilent, Santa Clara, CA) and the library concentrations were determined using a Kapa Library Quantification Kit (KAPA Biosystems). The libraries were pooled and loaded on MiSeq (Illumina) at a final concentration of 10 pM with 35% of PhiX spike-in and subjected to 300 bp paired-end sequencing.

### Bioinformatics for MiSeq data

For the sequence base quality control, we used FastQC and the program named PEAR to concatenate MiSeq reads. After that, we used the BWA-MEM program to align reads and generate an SAM alignment file, which was used as an input for the BAM-READCOUNT program to generate the genotype distribution and read coverage statistics for each base position along the target genomic region. For the structural variation analysis part, a customized analysis program was developed using the SAM alignment file to classify the reads into four classes, such as aligned reads with only deletion events, aligned reads with only insertion events, aligned reads with deletion and insertion events, and soft-clipped reads that likely contain insertions or deletions or both at the same time. For the first three classes of reads, based on the alignment CIGAR value for each aligned read, we computed coordinates for each structural variation. For example, a read with two deletions and two insertions will generate four records; each record contains the coordinates for the positions of the deletions or insertions as well as the genotype sequence. For the soft-clipped read class, we first realigned using the pairwise alignment function defined in the Biostrings R package to detect large insertions and deletions. Based on the detected structural variations, we constructed CIGAR values to generate the SAM file for the IGC visualization. Then, we applied the same strategy to generate the coordinates for each mutation, such as deletions and insertions. After obtaining mutation coordinates for each read, we applied different methods for deletion and insertion events. For each deletion event, which was identified by the deletion start and end positions, we grouped the reads that contained equal deletion start and end coordinates, and computed the number of reads in this group as the supporting evidence for such deletion events for this deletion range. For insertions, since the coordinates for each insertion are just one point, we classified the insertion sequence not only by the coordinates, but also by the length of insertion sequence. For deletions, we found the MH sequence flanking the two sides for each deletion range. For insertions, depending on the length of insertion sequence, such as for short ones of 10 bp, we searched the flanking regions and classified such an insertion sequence into three classes as template, snapback and undetermined. For longer insertion sequences, we searched for identification of the hits in the EJ5 sequence and the whole human genome sequence hg19, respectively. Reference sequences for high-throughput sequencing analysis are listed in Supplementary Table S1.

## RESULTS

### SBS3, ID6 and ID8 are enriched in *BRCA*-mutated cancers expressing high levels of wild-type *POLQ*

Since POLQ performs mutagenic end joining, the detection of POLQ-dependent unique mutation signatures may provide a means to monitor POLQ activity. It has been proposed that the COSMIC3 mutation signature may reflect POLQ mutagenesis (20,21). However, it has not yet been demonstrated whether *POLQ* status is associated with the enrichment of COSMIC3 in tumors.

**Figure 2.**
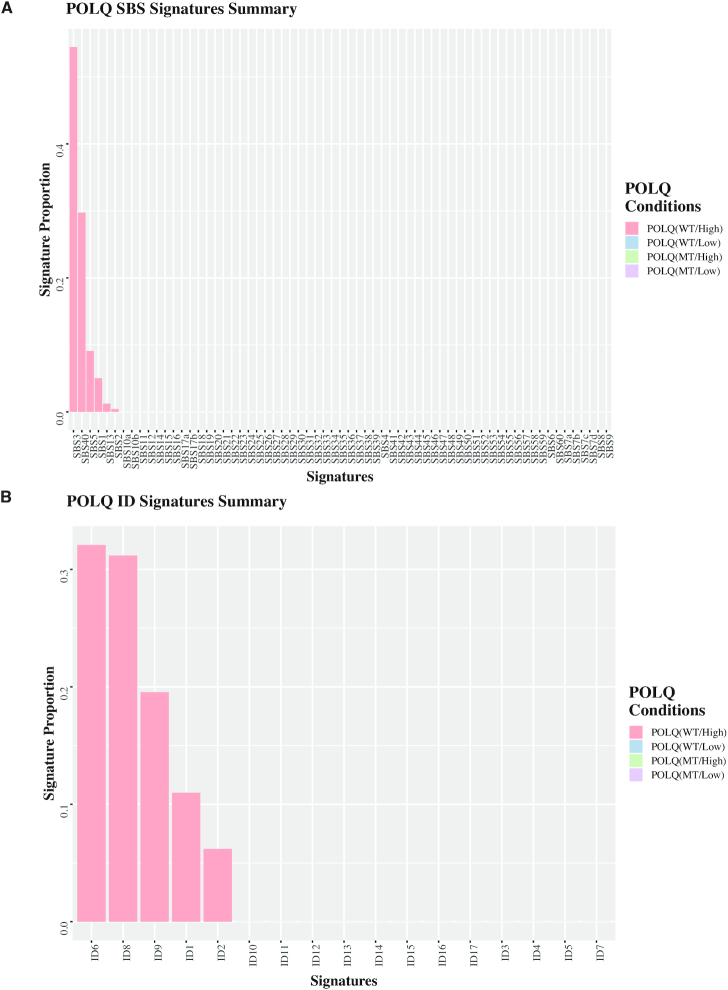
Mutation signatures enriched in *BRCA*-mutated tumors expressing wild-type or mutant form of *POLQ*. Proportions of 65 SBS (**A**) or 17 ID (**B**) signatures are determined according to *POLQ* mRNA expression level (high: top 33rd percentile; low: bottom 33rd percentile) and *POLQ* mutation status in *BRCA*-mutated cancers. The SBS and ID signature enrichment was detected only in *POLQ* WT/High but not in WT/Low, MT/High, MT/Low.

We analyzed enrichment of any of the 82 COSMIC signatures (65 SBS signatures or 17 ID signatures) according to *POLQ* mRNA expression level and *POLQ* mutation status in *BRCA*-mutated cancers (Supplementary Figure S1). *POLQ* expression was analyzed using RNA-seq data; the top and bottom 33% were considered as *POLQ* high-expression group and low-expression group, respectively. Most signatures were not associated with *POLQ* status. However, the proportion of SBS3 [a recently updated version 3 signature related to COSMIC3, a version 2 signature (17)] was enriched to 54% in the wild-type *POLQ* high-expression group in *BRCA*-mutated cancers (Figure 2A). The proportions of ID6 and ID8 were enriched by 32% and 31%, respectively (Figure 2B). SBS3 is one of the single base substitution signatures and is enriched in *BRCA*-mutated cancers (18). ID6 and ID8 are insertion/deletion signatures characterized by ≥5 bp deletions overlapping commonly with ≥2 and ≤3 bp MH, respectively (18). SBS3, ID6 and ID8 are signatures of MH-mediated end joining.

We further analyzed the relationship among those three mutational signatures and *POLQ* and *BRCA* status. SBS3, ID6 and ID8 were enriched more in *BRCA1/2*-mutated cancers expressing high levels of wild-type *POLQ* than in other conditions (Supplementary Figure S2A–C). ID6 strongly correlates with SBS3, but ID8 does not (18). We confirmed the correlation of SBS3 and ID6 by the Pearson correlation coefficient (*R* = 0.66, *P* < 2.2 × 10^−16^). The correlations of SBS3 and ID8 (*R* = 0.36, *P* < 2.2 × 10^−16^) and ID6 and ID8 (*R* = 0.31, *P* < 2.2 × 10^−16^) were weak (Supplementary Figure S2D). SBS3, ID6 and ID8 signatures were significantly enriched more than other types of mutation signatures in *BRCA1/2*-mutated cancers expressing wild-type *POLQ* (Supplementary Figure S2E). Other mutational signatures including SBS40, SBS5, ID9, ID1 and ID2 were not significantly enriched more in *BRCA1/2*-mutated cancers expressing wild-type *POLQ* than in other types of cancers (Supplementary Figure S3A and B).

Our data suggest that detection of the SBS3, ID6 and ID8 signatures may be useful for monitoring POLQ activity in tumors. However, these mutational signatures do not perfectly reflect POLQ mutagenesis. For example, ID6 includes deletions that overlap with any length of MH, while POLQ generally utilizes 2–6 bp MH for end joining (11,34). To improve the pipeline to monitor POLQ-dependent mutation signatures, it is important to enhance our understanding of features of POLQ mutagenesis.

### POLQ is involved in distal end joining

To directly explore the mechanism of POLQ action and mutagenesis, we analyzed two configurations of end joining, ‘proximal’ (where a DSB is directly joined) and ‘distal’ (joining of a break to a more distant break). A biological example of distal EJ is CSR of immunoglobulin heavy chain (*IgH*) genes. CSR is a ligation between two separated DSBs (distal EJ), following targeted introduction of DSBs into repetitive switch-region DNA elements in the *IgH* locus. In the mouse, POLQ sometimes introduces insertions during CSR (27). In the present experiments, the human *POLQ* gene was disrupted in DR-U2OS and EDS-7F2 cell lines by targeting the first exon with CRISPR/Cas9 (Supplementary Figure S4A). The DR-U2OS cell line carries a DR-GFP reporter system to monitor homologous recombination (HR) (29). The EDS-7F2 cell line carries pEJ5-GFP and pDsRed-I-SceI reporter cassettes to monitor distal EJ and chromosome translocation (30). The CRISPR/Cas9 disruptions introduced frameshift mutations in the open reading frame of *POLQ* (Supplementary Figure S4B). Immunoblotting with POLQ-specific antibody 1B1 (31) confirmed the absence of POLQ in the established *POLQ* knockout cell lines (Supplementary Figure S4C). The first exon sequences of *POLQ* in the wild-type allele and targeted alleles are shown in Supplementary Figure S4D.

**Figure 3.**
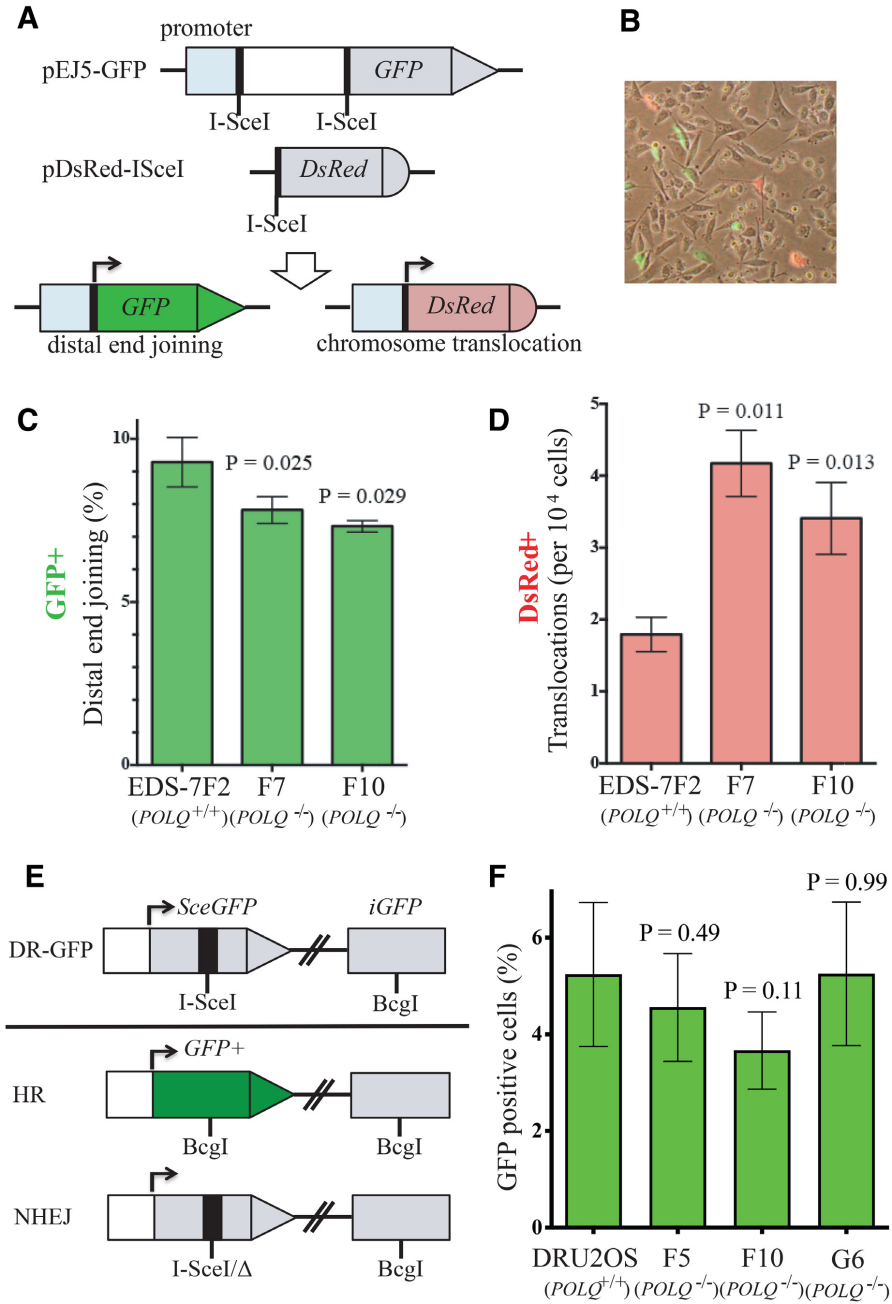
POLQ is involved in distal EJ. (**A**) Detection of distal EJ and chromosome translocation in human cells. The human bladder carcinoma cell line EDS-7F2 harbors a pEJ5-GFP construct on one chromosome and a pDsRed-I-SceI construct on another chromosome. For the break end-joining assay, the GFP gene and the DsRed genes are initially inactive due to the lack of a promoter but become activated following NHEJ between the I-SceI-induced DSBs. (**B**) Following infection with a retrovirus expressing I-SceI, the percentage of cells expressing GFP (distal EJ) or DsRed (chromosome translocation) was determined by flow cytometry. The frequencies of GFP+ (**C**) and DsRed+ (**D**) cells were determined in EDS-7F2 (7F2) and two *POLQ* knockout EDS-7F2 clones (F7 and F10). Error bars represent standard deviation of three separate experiments. The differences between 7F2 and *POLQ*^−/−^ cells (F7, F10) are statistically significant (*P* ≤ 0.05, unpaired *t*-tests). (**E**) *POLQ* knockout U2OS cell lines carry the recombination reporter DR-GFP integrated into the genome. *SceGFP* is a *GFP* gene that contains an I-SceI endonuclease site within the coding region. Cleavage of the I-SceI site *in vivo* and repair by HR directed by the downstream *iGFP* results in GFP+ cells. (**F**) HR in *POLQ*^+/+^ cells (DR-U2OS) and *POLQ*^−/−^ cells (F5, F10, G6) after I-SceI expression. The differences between DR-U2OS and F5, F10 and G6 cells are not statistically significant (*P* > 0.05, unpaired *t*-tests). This result was confirmed from four independent experiments.

To test the function of POLQ in distal EJ in human cells, we utilized the EDS-7F2 cell line. In EDS-7F2 cells, three specific I-SceI breaks can be induced by infection with a retrovirus expressing I-SceI and the efficiencies of distal EJ and chromosome translocations can be analyzed (30) (Figure 3A and B). The frequency of distal EJ (GFP+ cells) was significantly reduced in two *POLQ*-deficient clones (EDS-7F2 F7 and F10; Figure 3C). By contrast, the frequency of chromosome translocation (DsRed+ cells) was significantly increased in *POLQ*-deficient cells in this assay system (Figure 3D). The result is consistent with the reported function of mouse POLQ in suppressing the Myc-IgH translocation during CSR (27).

We found no statistically significant effect on HR by *POLQ* deletion in three distinct *POLQ*-deficient clones (Figure 3E and F) when we deleted the gene using CRISPR/Cas9 in a U2OS cell line with the DR-GFP assay system stably integrated in the genome to determine the *POLQ* knockout effect in human cells. Previous studies suggested that knocking down of human *POLQ* inhibits HR due to its interaction with RAD51 recombinase and inhibition of RAD51 loading onto DNA (7,8). However, like the human cells studied here *Polq*-deficient mice have normal levels of HR (35). A DR-GFP assay was used in all those experiments to measure gene conversion initiated by I-SceI-generated DSB (33).

### POLQ-dependent mutation signature is enriched in proximal end joining in NHEJ compromised cells

**Figure 4.**
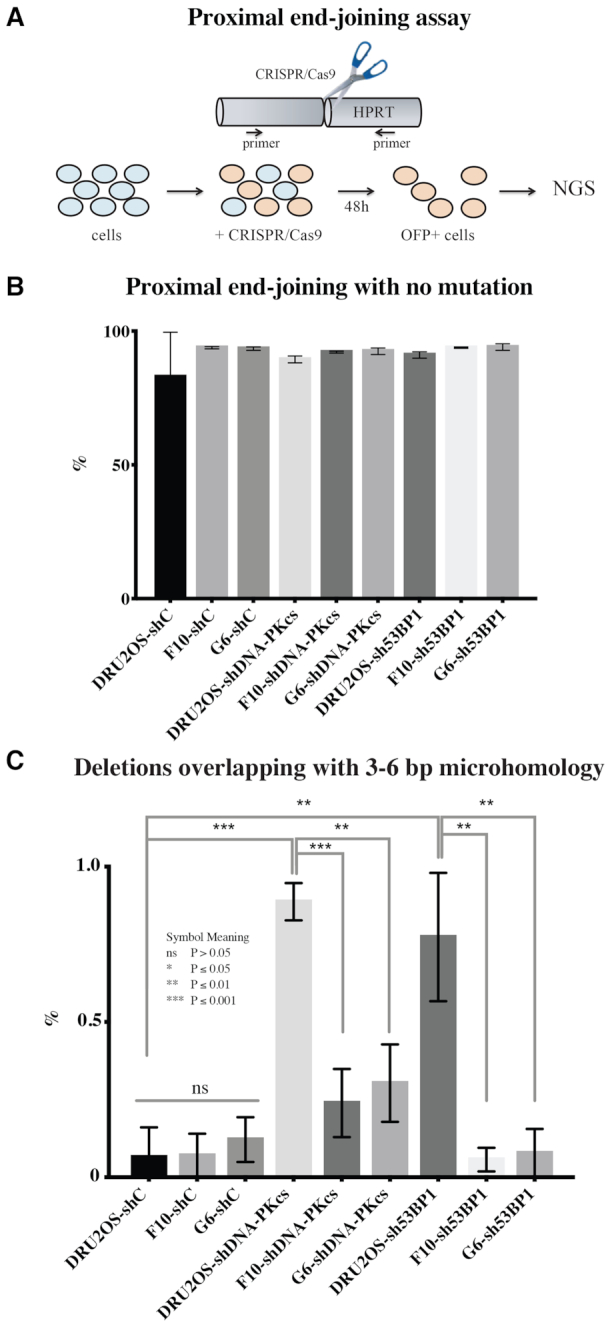
POLQ mutation signature accumulates in the absence of NHEJ in proximal EJ. (**A**) *HPRT* locus was targeted by CRISPR/Cas9. The cells expressing OFP, a co-expression marker, were isolated and the products of proximal EJ were characterized by target site amplification and high-throughput sequencing (NGS). The percentage of all proximal end-joining products with no mutation (**B**) and a POLQ mutation signature (deletion overlapping with 3–6 bp MH at the proximal end-joining site) (**C**). Unpaired *t*-tests were performed for statistic comparison. Parental DR-U2OS and *POLQ*^−/−^ clones (F10 and G6) are infected with shControl (shC), shDNA-PKcs or sh53BP1.

A better understanding of POLQ-dependent mutagenesis will improve the analysis of POLQ activity in tumors using genome sequencing data. In this context, we asked what genetic backgrounds influence POLQ mutagenesis in the process of joining DSBs that are introduced at one specific site (proximal EJ) in cultured human cells. We employed CRISPR/Cas9 to generate a specific DSB at exon 6 of the *HPRT* gene in the *POLQ*-defective and -proficient cells with stable knockdown of *DNA-PKcs* or *53BP1* using shRNA (Supplementary Figure S5A). Knockdown of *53BP1* in *POLQ*-defective cells is acutely toxic (20,21), but some clones survived and could be recovered and used in our analysis. End-joining products were isolated from the cells after introducing a specific DSB at the targeted locus by the CRISPR/Cas9 vector carrying OFP as a co-expression marker. We isolated OFP-positive cells as targeted cells by FACS. Genomic DNA from OFP-positive cells was isolated and the junction sequences were amplified by PCR, sequenced and analyzed (Figure 4A). The effects were determined by comparing the results from three independent experiments. Cutoff numbers of sequencing reads were determined based on Poisson distribution to exclude noise reads that occurred due to PCR and sequencing errors. The sequences found at less than the cutoff number (the Poisson 90%, 95% or 99% confidence interval depending on sample) were considered as background noise (Supplementary Table S2).

In our assay system, ∼90% of targeted loci were joined precisely (Figure 4B). We identified sequences that show ‘deletion with overlapping 3–6 bp of MH at the end-joining site’ as POLQ-mediated events. In human cells, end-joining junctions formed by NHEJ and TMEJ contain commonly 0–2 and 2–6 bp of MH, respectively (11,34). Those data match with biochemical activity of POLQ. POLQ requires a minimum of 2 bp and optimally 4 bp between a template and primer pair for efficient and processive DNA synthesis *in vitro* (36,37). We analyzed ∼10% of targeted loci that were imprecise end-joining products and found that unlike mouse cells (20), the frequency of this class of mutations was not influenced by disruption of *POLQ* alone in human cells. However, this signature increased significantly with *DNA-PKcs* or *53BP1* depletion. This increase was significantly reduced by *POLQ* deletion, indicating the mutation signature is due to POLQ activity (Figure 4C and Supplementary Table S3).

We also found that *POLQ* single deletion did not influence the frequency of imprecise end joining in human cells in the DR-GFP assay system in the absence of defects in additional DSB repair genes (Supplementary Figure S5B and C) when analyzed with a different method. Genomic DNA was isolated from pooled DR-U2OS cells following expression of I-SceI. In this assay system, if the I-SceI DSB is repaired by HR, the site becomes BcgI sensitive; if repaired by precise NHEJ, the site is still I-SceI sensitive; and if repaired by imprecise end joining (e.g. TMEJ), the sequence becomes resistant to I-SceI and BcgI (33).

Insertions were also identified at the junction sites. However, the majority of such events were insertion of a single T:A base pair. This is most likely a correct nucleotide insertion at the 1 bp staggered end, a preferred substrate of NHEJ (38). It has been reported that Cas9-catalyzed DNA cleavage produces 1 bp staggered ends rather than blunt ends (39). The 1 nt staggered ends generated by Cas9 are likely filled and joined by NHEJ. This activity was not influenced by POLQ, 53BP1 or DNA-PKcs. Although 53BP1 and DNA-PKcs influence NHEJ efficiency, those activities are not essential for end filling or end joining for the 1 nt staggered ends. 53BP1 inhibits resection of DNA breaks (40) and DNA-PKcs activates Artemis endonuclease activity (41) (Supplementary Figure S6AB and Supplementary Table S4).

### POLQ-dependent mutation signatures including MH-mediated templated insertions are enriched in distal end joining

POLQ-mediated nucleotide insertion was a rare event in our proximal end-joining assay system. We hypothesized that such events might be more common during distal EJ. POLQ introduces sequence insertions at end-joining sites during the distal end-joining process of CSR (27). Interestingly, induction of CSR by treatment of mouse B cells with IL-4 and lipopolysaccharide leads to upregulation of *Polq* in those cells, but not other genes including *Poln*, *Helq*, *Pold1* and *Haus3* (42). This is consistent with a role for POLQ in distal EJ.

**Figure 5.**
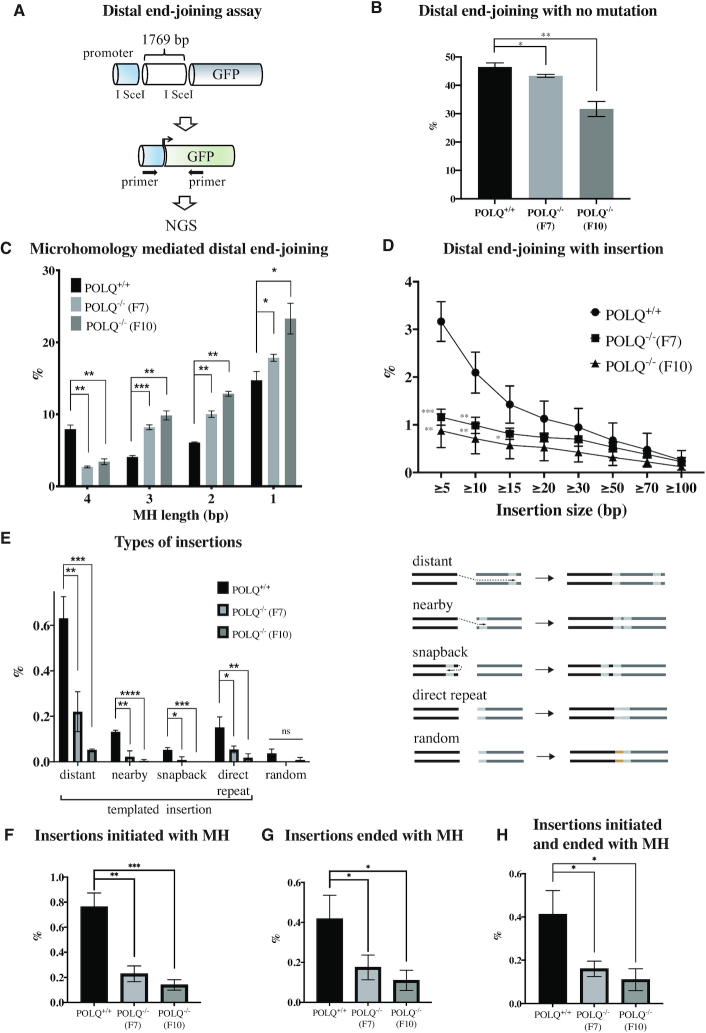
POLQ performs MH-mediated end joining and insertion in distal EJ. (**A**) Distal end-joining products isolated from GFP-positive cells were characterized by target site amplification and high-throughput sequencing (NGS). The percentage of end-joining products with no mutation (**B**) and different length MH (**C**). The percentage of different length of insertion (**D**), different types of insertion (**E**), insertions initiated with MH (**F**), insertions ended with MH (**G**) and insertions initiated and ended with MH (**H**). Unpaired *t*-tests were performed for statistical analysis (ns denotes *P* > 0.05; *P ≤ 0.05; **P ≤ 0.01; ***P ≤ 0.001).

We set out to test whether POLQ-dependent mutation signatures including templated insertions can be detected in nuclease-induced distal EJ. We isolated genomic DNA from GFP-positive *POLQ*^+/+^ or *POLQ*^−/−^ EDS-7F2 cells, in which two distal I-SceI breaks were joined (Figure 3C). PCR amplicons for the junction region were analyzed by high-throughput sequencing (Figure 5A). Cutoff numbers (the Poisson 99% confidence interval) were used to exclude noise reads that occurred due to the PCR and sequencing errors (Supplementary Table S5). We considered the 1769 bp deletion, detected when the two I-SceI break sites were simply joined, as end joining with no mutation. Mutations at the end-joining sites were identified in >50% of the sequences. Overall, a higher fraction of mutated sequences was found in *POLQ*^−/−^ cells (Figure 5B). In the deleted end-joining products, we found products overlapping with up to 4 bp of MH. The frequency of 4 bp MH was significantly reduced but the proportion of joining events with <4 bp MH was increased in *POLQ*^−/−^ clones. Products generated by Ku-mediated NHEJ [enriched for end joining with <4 bp MH (38,43)] likely increased in the absence of POLQ in this assay system. POLQ, which utilizes MH ranging between 2 and 6 bp (11,34), mediates the end joining with 4 bp MH (Figure 5C and Supplementary Table S6).

We next analyzed insertions generated at the end-joining sites. More insertions were identified in *POLQ*-proficient cells than *POLQ*-deficient cells. Insertions of ≥5 bp (up to 15 bp) occurred at significantly lower frequency in *POLQ* knockout cells (Figure 5D and Supplementary Table S7). We analyzed 5–15 bp insertions and categorized into five groups: distant, nearby, snapback, direct repeat and random. The ‘distant’ and ‘nearby’ categories are in *trans* insertions copied from template encoded >100 and <100 bp downstream or upstream from the end-joining junctions, respectively. The ‘snapback’ category refers to *cis* insertions copied from sequence existing upstream on the same strand. The ‘direct-repeat’ sequences are direct duplications of flanking DNA. Sequence insertions without an obvious template are ‘random’. We considered distant, nearby, snapback and direct-repeat insertions as templated insertions, and these were significantly reduced in *POLQ*^−/−^ cells (Figure 5E). We analyzed MH usage in distant, nearby and snapback insertions and found that those templated insertions were often initiated using MH by POLQ (Figure 5F) and that sequences at the end of the insertions are often used for MH-mediated end joining by POLQ (Figure 5G). Furthermore, templated insertions initiated and completed with MH were also significantly reduced in *POLQ*^−/−^ cells (Figure 5H and Supplementary Table S8). The origin of DNA synthesis was inferred based on the position of the templated sequence. For example, when the templated sequence was found downstream of the second I-SceI site (3530–3547), we considered the first I-SceI site (1758–1775) as the origin for the distant templated insertion. We considered that the second I-SceI site was the origin for the snapback templated insertion (Supplementary Tables S1 and S8).

**Figure 6.**
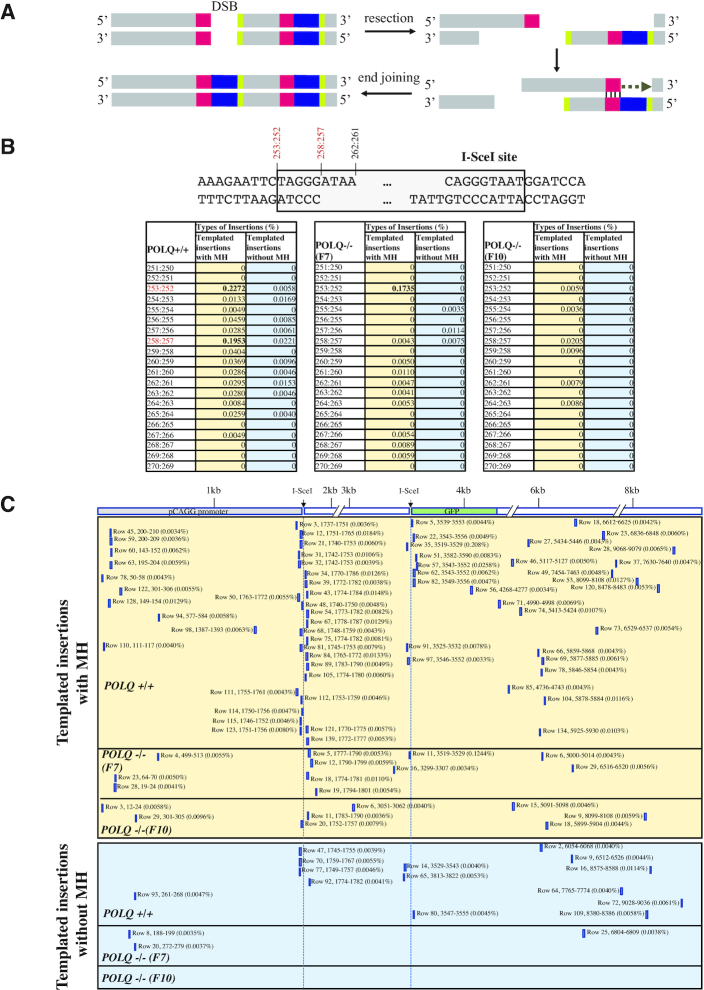
POLQ performs MH-mediated templated insertions. (**A**) A model of MH-mediated templated insertion. POLQ binds to the end of 3′ DNA tail following DNA resection and extends DNA using MH. Sometimes the sequence of the 3′ end of the extended DNA is used for MH-mediated end joining. Red: MH used to initiate DNA extension; black: copied sequence for insertion; green: 3′ end of the insertion that is sometimes used for MH-mediated end joining. (**B**) Hot spots for templated insertion (distant, nearby and snapback) initiated with or without MH. The number indicates the sequence position of the 429 bp PCR products for the intact allele (Supplementary Table S1). End-joined I-SceI sequence is 253:252–270:271. I-SceI cut site is 262:261. Percentage of each insertion is shown and the scores >0.1% are displayed in bold. (**C**) Positions of copied sequence for templated insertions (distant, nearby and snapback) are shown with percentage. Sequence with more than two possible copied sequences was excluded (Supplementary Figure S8). The number indicates the sequence position of the 9406 bp EJ5 reporter construct (Supplementary Table S1). The positions of the two I-SceI sites are 1758–1775 and 3530–3547. The row number indicates the sequence listed in Supplementary Table S8. Insertions initiated with and without MH are shown in top and bottom panels, respectively.

POLQ utilizes MH to initiate copying of sequences and also to join broken ends (Figure 6A). Templated insertions (5–15 bp) were often generated at the border of the I-SceI cleavage site (258:257) and at slightly further back from the break site (253:252; Figure 6B). By contrast, insertions at the original I-SceI break site 262:261 were found only rarely. One possible reason is that distal EJ occurs when individual breaks fail to undergo direct end joining. Such substrates, which were not repaired by NHEJ or HR, may be predominantly repaired by TMEJ. The position of MH-mediated insertions was not random, but instead specifically utilized embedded MH sequences. Templated insertions were copied from various regions of the inserted EJ5 construct DNA and their sequence locations are shown in Figure 6C.

## DISCUSSION

### SBS3, ID6 and ID8 reflect POLQ mutagenesis

In this study, we have demonstrated that specific COSMIC signatures, SBS3 and ID6 and ID8, are enriched in *BRCA*-mutated cancers that express high levels of wild-type *POLQ*. Those signatures may therefore be useful indicators of POLQ activity.

SBS3 is a single base substitution signature at all bases except C to D (D = A/G/T) substitutions at 5′-CG sequences, indicating that the signature is not associated with deamination of 5-methylcytosine (5-meC). 5-meC occurs predominantly at 5′-CG sequences in gene promoters. It is generally associated with transcriptional silencing. 5-meC residues in ssDNA are deaminated three times faster than C residues (44). Deamination of 5-meC results in the formation of thymine and hence of TG mispairs (45). Since C to T transitions at 5′-CG sequences are rare, SBS3 may be associated with DSBs at actively transcribing promoter regions that are not methylated. Indeed, Topoisomerase IIβ induces DSBs within promoter regions to facilitate the expression of a subset of genes (46). POLQ may process those DSBs especially in *BRCA*-mutated cancers. Interestingly, the majority of SBSs (68%) made by POLQ *in vitro* were generated when copying a template A or T. 5′-CG sequences were not POLQ hot spots (15). There is a resemblance between SBS3 and the POLQ base substitution signature.

ID6 is the most enriched signature among the ID signatures. It is a signature of MH-mediated end joining characterized by ≥5 bp deletions, commonly overlapping with ≥2 bp MH at breakpoint junctions. POLQ utilizes 2–6 bp MH for end joining (11,34); thus, ID6 closely reflects POLQ mutagenesis. ID8 is the second most enriched signature. ID8 encompasses deletions with short MH (commonly ≤3 bp), which are likely end-joining products by NHEJ. Although it is not an ideal substrate, POLQ can extend DNA from a primer annealed with 2 bp MH (36,37). ID8 thus appears to be the mixture of signatures generated by NHEJ and TMEJ.

Other mutational signatures including SBS40, SBS5, ID9, ID1 and ID2 were also enriched in *BRCA1/2*-mutated cancers expressing high levels of wild-type *POLQ*. However, they were not significantly enriched more in *BRCA1/2*-mutated cancers expressing wild-type *POLQ* than in other types of cancers (Supplementary Figure S3). SBS40 and SBS5 are correlated with age of cancer diagnosis and contribute to multiple types of cancer (18). The association between those signatures and POLQ activity is unknown. ID9, ID1 and ID2 are signatures of single base insertion and deletion at homopolymeric runs (18). This matches with the biochemical observation that POLQ adds and deletes single nucleotides during DNA replication of long mononucleotide tracts at particularly high rates (15). POLQ activity might therefore contribute to ID9, ID1 and ID2.

### POLQ has a role in mediating distal end joining in DSB repair

The major function for POLQ is in the defense against DSBs. It has been proposed that POLQ functions in suppressing HR by modulating RAD51 loading (7,8). However, this is unlikely because RAD51 is orders of magnitude more abundant than POLQ. Increased RAD51 foci in *POLQ*-defective cells may be a sign of aborted recombination events. Some breaks (e.g. those with resection) are best repaired by TMEJ, and if TMEJ is absent, HR cannot successfully process them. In a DR-GFP assay in U2OS cells, HR was reported to increase ∼2-fold after siRNA-mediated *POLQ* knockdown (7,8). However, HR efficiency measured by the DR-GFP assay in mouse cells was not affected after knocking out *Polq* (35). In this study, we knocked out the *POLQ* gene in the U2OS DR-GFP (DR-U2OS) cells and also found that HR was not affected by *POLQ* single deletion in human cells.

Our data reveal a role for POLQ in distal EJ. Distal EJ is reduced in the absence of POLQ (Figure 3) and POLQ-dependent mutation signatures are enriched in distal EJ (Figure 5). On the other hand, the POLQ-dependent mutation signature in proximal EJ increases only when the NHEJ pathway is perturbed (Figure 4). The results indicate that proximal EJ is predominantly processed by NHEJ, but distal EJ is often processed by TMEJ. This may explain enrichment of POLQ-dependent templated insertions during CSR, a physiological example of distal EJ (27). Translocations joining parts of different chromosomes are also examples of distal EJ, and are another candidate for future analysis for the presence of a TMEJ signature at translocation sites.

POLQ uses resected DNA ends as substrates, but those resected ends may be modified to allow for repair by NHEJ in the absence of POLQ. For example, CTC1–STN1–TEN1 (CST)–Polα fills in such resected DNA ends to promote NHEJ (47). NHEJ usually performs end joining with <4 bp MH (38), which could account for the increase in distal end-joining products with <4 bp MH in the absence of POLQ (Figure 5C).

TMEJ is an important alternative for other DSB repair pathways, NHEJ and HR (Figure 1). TMEJ is protective for genome stability, in the sense that an absence of POLQ leads to large deletions in some conditions, such as an NHEJ-deficient background (11,19). Our data reveal a role for POLQ in preventing chromosome translocations. The use of TMEJ results in an increase in short deletions and insertions, but it helps prevent catastrophic large deletions and chromosome rearrangements.

In summary, we have identified here several POLQ-associated mutation signatures. These are COSMIC signatures SBS3 and ID6, deletions with 3–6 bp MH arising from proximal EJ in the absence of NHEJ and templated insertions arising from distal EJ. Currently, genomic sequencing of tumor biopsies is increasingly done with the intention of informing treatment decisions. Tumors enriched for these POLQ-associated signatures may have lost functional HR or NHEJ, and subsequently may be hypersensitive to POLQ inhibition and DSB-inducing therapies. The better understanding of POLQ-induced mutation signatures as reported here will help to develop more effective pipelines to detect POLQ-dependent mutation signatures in genome sequencing data.

## Supplementary Material

zcaa017_Supplemental_Files
