# Supplementary material for: Defining the mutation signatures of DNA polymerase θ in cancer genomes
Source: NAR Cancer. 2020 Aug 27;2(3):zcaa017. doi: 10.1093/narcan/zcaa017 (PMC7454005; doi:10.1093/narcan/zcaa017)
Supplement: zcaa017_Supplemental_Files [file zcaa017_supplemental_files.zip › Supplementary Material_NAR Cancer.docx]

**SUPPLEMENTARY INFORMATIONS**

**Supplementary Fig. 1: POLQ-mediated mutational signature analysis workflow.**

**Supplementary Fig. 2: Enrichment of SBS3, ID6 and ID8 signatures in *BRCA* mutated cancers expressing wild-type *POLQ*.** Proportions of SBS3 (A), ID6 (B) and ID8 (C) are determined according to *POLQ* status (NOC; no conditional, WT; wild-type, MT; mutation), *POLQ* mRNA expression level (NOC, high; top 33^rd^ percentile, low; bottom 33^rd^ percentile), and *BRCA1* or *BRCA2* status (NOC; no conditional, WT; wild-type, MT; mutation). (D) The Pearson correlation coefficient measures correlation of SBS3, ID6, and ID8. (E) Statistical test verification for enrichment of SBS3, ID6 and ID8 in *BRCA1/2* mutated and *POLQ* wild-type samples. The Wilcoxon signed-rank test was used for statistical comparison. We further adjusted p-value by the Bonferroni method for stringent statistical significance.

**Supplementary Fig. 3: Other mutational signatures (SBS40, SBS5, ID9, ID1, ID2) and status of *BRCA* and *POLQ*.** (A) Proportions of SBS40, SBS5, ID9, ID1, ID2 are determined according to *POLQ* status (NOC; no conditional, WT; wild-type, MT; mutation), *POLQ* mRNA expression level (NOC, high; top 33^rd^ percentile, low; bottom 33^rd^ percentile), and *BRCA1* or *BRCA2* status (NOC; no conditional, WT; wild-type, MT; mutation). (B) Statistical test verification for enrichment of SBS40, SBS5, ID9, ID1, and ID2 in *BRCA1/2* mutated and *POLQ* wild-type samples. The Wilcoxon signed-rank test was used for statistical comparison. We further adjusted p-value by the Bonferroni method for stringent statistical significance.

**Supplementary Fig. 4:** **Inactivation of POLQ in human cells** (A) Both copies of POLQ in the human cell lines were targeted in the first exon by CRISPR/Cas9, producing deletions or insertions that yield early truncating frameshifts. The *POLQ* specific gRNA binding site is shown in red. (B) Targeting POLQ in the DR-U2OS cell line established the POLQ disrupted DR-U2OS cell lines F5 with the same 2 nt deletion in both alleles changing the open reading frame (ORF) after amino acid 22 with a stop codon arising after amino acid 24; F10 with the same 463 nt insertion in both alleles changing the ORF after amino acid 23 with a stop codon arising after amino acid 66; and G6 with the same 23 nt insertion in both alleles changing the ORF after amino acid 23 and creating stop codon after amino acid 46. Targeting POLQ in the EDS-7F2 cell line established the POLQ disrupted EDS-7F2 cell lines F7 with 1 nt deletion in one allele and 530 nt insertion in another allele changing the ORF after amino acid 22 and 24 respectively with a stop codon after amino acid 38 and 138, respectively; and F10 with 2 nt deletion in one allele and 10 nt deletion in another allele changing the ORF after amino acid 22 and creating stop codon after amino acid 24 and 35, respectively. The open reading frame of the wild-type allele is 7767 bp and encodes 2618 amino acids. Bold type indicates unaltered sequence and * indicates a stop codon. (C) Immunoblots show absence of POLQ in established POLQ knock out cell lines. (D) Genomic DNA sequence of POLQ knockout cell lines. The first exon sequence of POLQ in wild-type allele and targeted alleles. Lowercase indicates 5’UTR sequence and uppercase indicates open reading frame of POLQ in wild-type allele. Underlined sequence is the targeted sequence where gRNA binds. Deleted sequence is shown as *, inserted sequence is shown with bold letter.

**Supplementary Fig. 5:** **shRNA-mediated knockdown and the frequency of imprecise end-joining products in *POLQ* knockout human cells** (A) immunoblot showing efficacy of shRNA-mediated knockdown of DNA-PKcs and 53BP1 in *POLQ*^+/+^ and two *POLQ*^-/-^ (F10 and G6) cells. shControl (shC) served as a control and α-Tubulin and Vinculin as loading control. (B) A representative gel result for I-SceI site loss assay. Samples transfected with I-SceI are indicated with + I-SceI. The genomic region surrounding the I-SceI break site was amplified by PCR. The 650 bp band in the I-SceI + BcgI digest represents amplified product that has undergone repair by imprecise EJ, the 500 bp band represents products repaired by precise EJ or homologous recombination (C) Quantified PCR products that were resistant to I-SceI and BcgI (Imprecise EJ) from DR-U2OS and *POLQ*^-/-^ DR-U2OS cell lines (F5, F10, G6) from three independent experiments. Because the 500 bp band is weaker than the 650 bp band due to its reduced length, it is necessary to include a 650/500 correction. The formula is: Percent imprecise EJ = 100 x 650 bp band / (650 bp band + [650/500] x 500 bp band). Unpaired t-tests were performed for statistic comparison.

**Supplementary Fig. 6:** **POLQ-independent specific one nucleotide insertion is evidence of 1 nt staggered ends generated by Cas9.** (A) Illustration shows the CRISPR/Cas9 target sequence with a box indicating PAM sequence, triangles indicating cut sites. After a break, nucleotides shown in red were incorporated and ligated. (B) Possibility of T (and A) insertion at the break site. It was rare to find incorporation of other nucleotides at the site. This activity was POLQ-independent. Unpaired t-tests were performed for statistic comparison.
