# Supplementary material for: Defining the mutation signatures of DNA polymerase θ in cancer genomes
Source: NAR Cancer. 2020 Aug 27;2(3):zcaa017. doi: 10.1093/narcan/zcaa017 (PMC7454005; doi:10.1093/narcan/zcaa017)
Supplement: zcaa017_Supplemental_Files [file zcaa017_supplemental_files.zip › Supplementary Table 1.docx]

**Supplementary Table1.** Reference sequences used for high-throughput sequencing analysis.

The reference sequence for the proximal end-joining (HPRT ex6, CRISPR/Cas9-targeted sequence is underlined): 5’TCTTACTGCTTGCTGAGGGCCAGATGATATAGATTCCAGAATATCTCCATGTAGATTTTGGTGAGAATTACTGTGCTGAAAAGAATGACAGTATTGCAGTTATACATGGGGGTTTTGGTACTTTATATTGTGACTCTGAATTTAAAGCTATGCAATGTCTTCTTTTTTGAAAGGATATAATTGACACTGGCAAAACAATGCAGACTTTGCTTTCCTTGGTCAGGCAGTATAATCCAAAGATGGTCAAGGTCGCAAGGTATGTATGACATTTTGACACAGAATATTTTCCTCATTTGAAGGGGGATTAAGTGATTGCTTCTTTTTAAGGATAAATGTTTTCAACTGTCATTTTATCTTCGAAAAGTAATGTAATCTCATATAAGACTTAAGATATAATCCTTTTAAATAATTTTGTCATGTGTTAATAAAGCTCATAATTACAGTCACTTCCTTGCCTAATATTAACATTTGGTTTTTCAGCATGCTAATTATATCAGTTTGTCCTGAATAGCATGGCAGAGGATTTTGGGCCCCCTTGCAAAATTA

The reference sequence for the distal end-joining (GFP-positive EDS 7F2, I-SceI site is underlined):

5’AGGAAGGAAATGGGCGGGGAGGGCCTTCGTGCGTCGCCGCGCCGCCGTCCCCTTCTCCCTCTCCAGCCTCGGGGCTGTCCGCGGGGGGACGGCTGCCTTCGGGGGGGACGGGGCAGGGCGGGGTTCGGCTTCTGGCGTGTGACCGGCGGCTCTAGAGCCTCTGCTAACCATGTTCATGCCTTCTTCTTTTTCCTACAGCTCCTGGGCAACGTGCTGGTTATTGTGCTGTCTCATCATTTTGGCAAAGAATTCTAGGGATAACAGGGTAATGGATCCACCGGTCGCCACCATGGTGAGCAAGGGCGAGGAGCTGTTCACCGGGGTGGTGCCCATCCTGGTCGAGCTGGACGGCGACGTAAACGGCCACAAGTTCAGCGTGTCCGGCGAGGGCGAGGGCGATGCCACCTACGGCAAGCTGACCCTGAAGTT

The reference sequence for the sequence insertion in distal end-joining (original EJ5 construct, I-SceI sites are underlined):

5’ATTATTGAAGCATTTATCAGGGTTATTGTCTCATGAGCGGATACATATTTGAATGTATTTAGAAAAATAAACAAATAGGGGTTCCGCGCACATTTCCCCGAAAAGTGCCACCTGGTCGACATTGATTATTGACTAGTTATTAATAGTAATCAATTACGGGGTCATTAGTTCATAGCCCATATATGGAGTTCCGCGTTACATAACTTACGGTAAATGGCCCGCCTGGCTGACCGCCCAACGACCCCCGCCCATTGACGTCAATAATGACGTATGTTCCCATAGTAACGCCAATAGGGACTTTCCATTGACGTCAATGGGTGGACTATTTACGGTAAACTGCCCACTTGGCAGTACATCAAGTGTATCATATGCCAAGTACGCCCCCTATTGACGTCAATGACGGTAAATGGCCCGCCTGGCATTATGCCCAGTACATGACCTTATGGGACTTTCCTACTTGGCAGTACATCTACGTATTAGTCATCGCTATTACCATGGGTCGAGGTGAGCCCCACGTTCTGCTTCACTCTCCCCATCTCCCCCCCCTCCCCACCCCCAATTTTGTATTTATTTATTTTTTAATTATTTTGTGCAGCGATGGGGGCGGGGGGGGGGGGGGCGCGCGCCAGGCGGGGCGGGGCGGGGCGAGGGGCGGGGCGGGGCGAGGCGGAGAGGTGCGGCGGCAGCCAATCAGAGCGGCGCGCTCCGAAAGTTTCCTTTTATGGCGAGGCGGCGGCGGCGGCGGCCCTATAAAAAGCGAAGCGCGCGGCGGGCGGGAGTCGCTGCGTTGCCTTCGCCCCGTGCCCCGCTCCGCGCCGCCTCGCGCCGCCCGCCCCGGCTCTGACTGACCGCGTTACTCCCACAGGTGAGCGGGCGGGACGGCCCTTCTCCTCCGGGCTGTAATTAGCGCTTGGTTTAATGACGGCTCGTTTCTTTTCTGTGGCTGCGTGAAAGCCTTAAAGGGCTCCGGGAGGGCCCTTTGTGGAGCGCTGCGGGCGCGGCGCGGGGCTTTGTGCGCTCCGCGTGTGCGCGAGGGGAGCGCGGCCGGGGGCGGTGCCCCGCGGTGCGGGGGGGCTGCGAGGGGAACAAAGGCTGCGTGCGGGGTGTGTGCGTGGGGGGGTGAGCAGGGGGTGTGGGCGCGGCGGTCGGGCTGTAACCCCCCCCTGCACCCCCCTCCCCGAGTTGCTGAGCACGGCCCGGCTTCGGGTGCGGGGCTCCGTGCGGGGCGTGGCGCGGGGCTCGCCGTGCCGGGCGGGGGGTGGCGGCAGGTGGGGGTGCCGGGCGGGGCGGGGCCGCCTCGGGCCGGGGAGGGCTCGGGGGAGGGGCGCGGCGGCCCCGGAGCGCCGGCGGCTGTCGAGGCGCGGCGAGCCGCAGCCATTGCCTTTTATGGTAATCGTGCGAGAGGGCGCAGGGACTTCCTTTGTCCCAAATCTGGCGGAGCCGAAATCTGGGAGGCGCCGCCGCACCCCCTCTAGCGGGCGCGGGCGAAGCGGTGCGGCGCCGGCAGGAAGGAAATGGGCGGGGAGGGCCTTCGTGCGTCGCCGCGCCGCCGTCCCCTTCTCCCTCTCCAGCCTCGGGGCTGTCCGCGGGGGGACGGCTGCCTTCGGGGGGGACGGGGCAGGGCGGGGTTCGGCTTCTGGCGTGTGACCGGCGGCTCTAGAGCCTCTGCTAACCATGTTCATGCCTTCTTCTTTTTCCTACAGCTCCTGGGCAACGTGCTGGTTATTGTGCTGTCTCATCATTTTGGCAAAGAATTCTAGGGATAACAGGGTAATAATTCTACCGGGTAGGGGAGGCGCTTTTCCCAAGGCAGTCTGGAGCATGCGCTTTAGCAGCCCCGCTGGGCACTTGGCGCTACACAAGTGGCCTCTGGCCTCACACATTCCACATCCACCGGTAGGCGCCAACCGGCTCCGTTCTTTGGTGGCCCCTTCGCGCCACCTTCTACTCCTCCCCTAGTCAGGAAGTTCCCCCCCGCCCCGCAGCTCGCGTCGTGCAGGACGTGACAAATGGAAGTAGCACGTCTCACTAGTCTCGTGCAGATGGACAGCACCGCTGAGCAATGGAAGCGGGTAGGCCTTTGGGGCAGCGGCCAATAGCAGCTTTGCTCCTTCGCTTTCTGGGCTCAGAGGCTGGGAAGGGGTGGGTCCGGGGGCGGGCTCAGGGGCGGGCTCAGGGGCGGGGCGGGCGCCCGAAGGTCCTCCGGAGGCCCGGCATTCTGCACGCTTCAAAAGCGCACGTCTGCCGCGCTGTTCTCCTCTTCCTCATCTCCGGGCCTTTCGACCGATCCAGCCGCCACCATGACCGAGTACAAGCCCACGGTGCGCCTCGCCACCCGCGACGACGTCCCCCGGGCCGTACGCACCCTCGCCGCCGCGTTCGCCGACTACCCCGCCACGCGCCACACCGTCGACCCGGACCGCCACATCGAGCGGGTCACCGAGCTGCAAGAACTCTTCCTCACGCGCGTCGGGCTCGACATCGGCAAGGTGTGGGTCGCGGACGACGGCGCCGCGGTGGCGGTCTGGACCACGCCGGAGAGCGTCGAAGCGGGGGCGGTGTTCGCCGAGATCGGCCCGCGCATGGCCGAGTTGAGCGGTTCCCGGCTGGCCGCGCAGCAACAGATGGAAGGCCTCCTGGCGCCGCACCGGCCCAAGGAGCCCGCGTGGTTCCTGGCCACCGTCGGCGTCTCGCCCGACCACCAGGGCAAGGGTCTGGGCAGCGCCGTCGTGCTCCCCGGAGTGGAGGCGGCCGAGCGCGCCGGGGTGCCCGCCTTCCTGGAGACCTCCGCGCCCCGCAACCTCCCCTTCTACGAGCGGCTCGGCTTCACCGTCACCGCCGACGTCGAGTGCCCGAAGGACCGCGCGACCTGGTGCATGACCCGCAAGCCCGGTGCCTGACGCCCGCCCCACGACCCGCAGCGCCCGACCGAAAGGAGCGCACGACCCCATGGCTCCGACCGAAGCCGACCCGGGCGGCCCCGCCGACCCCGCACCCGCCCCCGAGGCCCACCGCGGGGGACACACCGAACACGCCGACCCTGCTGAACACGCGGCGCAGTTCGGTGCCCAGGAGCGGATCGAAATTGATGATCTATTAAACAATAAAGATGTCCACTAAAATGGAAGTTTTTCCTGTCATACTTTGTTAAGAAGGGTGAGAACAGAGTACCTACATTTTGAATGGAAGGATTGGAGCTACGGGGGTGGGGGTGGGGATTAGATAAATGCCTGCTCTTTACTGAAGGCTCTTTACTATTGCTTTATGATAATGTTTCATAGTTGGATATCATAATTTAAACAAGCAAAACCAAATTAAGGGCCAGCTCATTCCTCCCACTCATGATCTATAGATCTATAGATCTCTCGTGGGATCATTGTTTTTCTCTTGATTCCCACTTTGTGGTTCTAAGTACTGTGGTTTCCAAATGTGTCAGTTTCATAGCCTGAAGAACGAGATCAGCAGCCTCTGTTCCACATACACTTCATTCTCAGTATTGTTTTGCCAAGTTCTAATTCCATCAGAAGCTTAGGGATAACAGGGTAATGGATCCACCGGTCGCCACCATGGTGAGCAAGGGCGAGGAGCTGTTCACCGGGGTGGTGCCCATCCTGGTCGAGCTGGACGGCGACGTAAACGGCCACAAGTTCAGCGTGTCCGGCGAGGGCGAGGGCGATGCCACCTACGGCAAGCTGACCCTGAAGTTCATCTGCACCACCGGCAAGCTGCCCGTGCCCTGGCCCACCCTCGTGACCACCCTGACCTACGGCGTGCAGTGCTTCAGCCGCTACCCCGACCACATGAAGCAGCACGACTTCTTCAAGTCCGCCATGCCCGAAGGCTACGTCCAGGAGCGCACCATCTTCTTCAAGGACGACGGCAACTACAAGACCCGCGCCGAGGTGAAGTTCGAGGGCGACACCCTGGTGAACCGCATCGAGCTGAAGGGCATCGACTTCAAGGAGGACGGCAACATCCTGGGGCACAAGCTGGAGTACAACTACAACAGCCACAACGTCTATATCATGGCCGACAAGCAGAAGAACGGCATCAAGGTGAACTTCAAGATCCGCCACAACATCGAGGACGGCAGCGTGCAGCTCGCCGACCACTACCAGCAGAACACCCCCATCGGCGACGGCCCCGTGCTGCTGCCCGACAACCACTACCTGAGCACCCAGTCCGCCCTGAGCAAAGACCCCAACGAGAAGCGCGATCACATGGTCCTGCTGGAGTTCGTGACCGCCGCCGGGATCACTCTCGGCATGGACGAGCTGTACAAGTAAAGCGGGGGATGCAGAAATTGATGATCTATTAAACAATAAAGATGTCCACTAAAATGGAAGTTTTTCCTGTCATACTTTGTTAAGAAGGGTGAGAACAGAGTACCTACATTTTGAATGGAAGGATTGGAGCTACGGGGGTGGGGGTGGGGTGGGATTAGATAAATGCCTGCTCTTTACTGAAGGCTCTTTACTATTGCTTTATGATAATGTTTCATAGTTGGATATCATAATTTAAACAAGCAAAACCAAATTAAGGGCCAGCTCATTCCTCCCACTCATGATCTATAGATCTATAGATCTCTCGTGGGATCATTGTTTTTCTCTTGATTCCCACTTTGTGGTTCTAAGTACTGTGGTTTCCAAATGTGTCAGTTTCATAGCCTGAAGAACGAGATCAGCAGCCTCTGTTCCACATACACTTCATTCTCAGTATTGTTTTGCCAAGTTCTAATTCCATCAGAAGCTGGTCGACTCTAGCACCTTTTTTTTTTTTTTTTTTTTTTACTCCCCAAAGTTTGTAAACAGGAATCACGTGGTCTGAAACCAGCCCTGCAACTGTACCTTTCCAGTCTAGGGAGTGGGAGAGTGCAGTGTCTATGCCTTGCTTGCATAGAACCCGGAGGAAAGCACACCCTGAGTTAGTTGTGTATAAAGGCCCTTACCTGGGCCCGGTCTAGATGAGACTGGTGTGGCAGGGCCTGGGGTCCTTGTAGTTAAAGGCTAACCCACTGAGTGGCCTCTAGACTTGAGCTTTTCGGTGGCTTTATTTGTCTTTGGGCCGATGTGTAAGGGATTTGGGTTTAGGGTTTTTGGGCTCCCAGAGAGCTACCATATCATTTCTTCATTTTCTGTCTGGACCAGAGAGGCAGAGATGGAGAGAAAAAAAAATATGGTTGTTTTAAAGTCAAATCTGCCTGTCTGCCTCCTGAGAGCTGGGATTAAAGGCATGTGCCACCAAACCCGAGCCAGGCAGTGTCTTTATTAAAAAATATTTGAAAACCTAGTGGAGGTGGTGGTTCTTGCTTTTAATCCAGCCCTTGGGAGGTAGAGGTAGGCAGATCTCCGAGTTCAAGGCCAGCCTGGCCTACAGAGTCACTTCCAGAGCTACACAGAGAAACTTTGGAGGAGAAAAAAAAAATTAAAAGAAGCGGCTCAGACTAGCTGCTTTGTTAACTTTTTTTTTGCTAATGCTGGTTTCTTTCTATTCCCTCTGCTGGTAGGGACCCGAGTGTACAGTCCTCCAGAGTGGATTCGCTACCATCGCTACCACGGAGGTGGCCATTAAGCACGTGGAGAAGGACCGGATTTCCGATTGGGGAGAACTGGTGAGTGAGCCGGAGCCAGAGACCCTCTCATGGGTGGGTGGGTGGCGACGGGCGTGCCGGAACCGGCGCGCTAACCCTCGCCCTCACTCTGCAGCCCAATGGCACCCGAGTGCCCATGGAAGTGGTCCTGTTGAAGAAGGTGAGCTCCAGGTCGGCAGAGAGCTCACGTCATGCGGCCGCTGTGGCACTTTTCGGGGAAATGTGCGCGGAACCCCTATTTGTTTATTTTTCTAAATACATTCAAATATGTATCCGCTCATGAGACAATAACCCTGATAAATGCTTCAATAATATTGAAAAAGGAAGAGTATGAGTATTCAACATTTCCGTGTCGCCCTTATTCCCTTTTTTGCGGCATTTTGCCTTCCTGTTTTTGCTCACCCAGAAACGCTGGTGAAAGTAAAAGATGCTGAAGATCAGTTGGGTGCACGAGTGGGTTACATCGAACTGGATCTCAACAGCGGTAAGATCCTTGAGAGTTTTCGCCCCGAAGAACGTTTTCCAATGATGAGCACTTTTAAAGTTCTGCTATGTGGCGCGGTATTATCCCGTATTGACGCCGGGCAAGAGCAACTCGGTCGCCGCATACACTATTCTCAGAATGACTTGGTTGAGTACTCACCAGTCACAGAAAAGCATCTTACGGATGGCATGACAGTAAGAGAATTATGCAGTGCTGCCATAACCATGAGTGATAACACTGCGGCCAACTTACTTCTGACAACGATCGGAGGACCGAAGGAGCTAACCGCTTTTTTGCACAACATGGGGGATCATGTAACTCGCCTTGATCGTTGGGAACCGGAGCTGAATGAAGCCATACCAAACGACGAGCGTGACACCACGATGCCTGTAGCAATGGCAACAACGTTGCGCAAACTATTAACTGGCGAACTACTTACTCTAGCTTCCCGGCAACAATTAATAGACTGGATGGAGGCGGATAAAGTTGCAGGACCACTTCTGCGCTCGGCCCTTCCGGCTGGCTGGTTTATTGCTGATAAATCTGGAGCCGGTGAGCGTGGGTCTCGCGGTATCATTGCAGCACTGGGGCCAGATGGTAAGCCCTCCCGTATCGTAGTTATCTACACGACGGGGAGTCAGGCAACTATGGATGAACGAAATAGACAGATCGCTGAGATAGGTGCCTCACTGATTAAGCATTGGTAACTGTCAGACCAAGTTTACTCATATATACTTTAGATTGATTTAAAACTTCATTTTTAATTTAAAAGGATCTAGGTGAAGATCCTTTTTGATAATCTCATGACCAAAATCCCTTAACGTGAGTTTTCGTTCCACTGAGCGTCAGACCCCGTAGAAAAGATCAAAGGATCTTCTTGAGATCCTTTTTTTCTGCGCGTAATCTGCTGCTTGCAAACAAAAAAACCACCGCTACCAGCGGTGGTTTGTTTGCCGGATCAAGAGCTACCAACTCTTTTTCCGAAGGTAACTGGCTTCAGCAGAGCGCAGATACCAAATACTGTCCTTCTAGTGTAGCCGTAGTTAGGCCACCACTTCAAGAACTCTGTAGCACCGCCTACATACCTCGCTCTGCTAATCCTGTTACCAGTGGCTGCTGCCAGTGGCGATAAGTCGTGTCTTACCGGGTTGGACTCAAGACGATAGTTACCGGATAAGGCGCAGCGGTCGGGCTGAACGGGGGGTTCGTGCACACAGCCCAGCTTGGAGCGAACGACCTACACCGAACTGAGATACCTACAGCGTGAGCATTGAGAAAGCGCCACGCTTCCCGAAGGGAGAAAGGCGGACAGGTATCCGGTAAGCGGCAGGGTCGGAACAGGAGAGCGCACGAGGGAGCTTCCAGGGGGAAACGCCTGGTATCTTTATAGTCCTGTCGGGTTTCGCCACCTCTGACTTGAGCGTCGATTTTTGTGATGCTCGTCAGGGGGGCGGAGCCTATGGAAAAACGCCAGCAACGCGGCCTTTTTACGGTTCCTGGCCTTTTGCTGGCCTTTTGCTCACATGTTCTTTCCTGCGTTATCCCCTGATTCTGTGGATAACCGTATTACCGCCTTTGAGTGAGCTGATACCGCTCGCCGCAGCCGAACGACCGAGCGCAGCGAGTCAGTGAGCGAGGAAGCGGAAGAGCGCCCAATACGCAAACCGCCTCTCCCCGCGCGTTGGCCGATTCATTAATGCAGGTTAACCTGGCTTATCGAAATTAATACGACTCACTATAGGGAGACCGGCAGATCTGATCCGCTCGAGCGGATCATCGCCGGGTGAGTGTCCCCACCCCACCCTGGGCGCGCGGGTCGTTGGGACGCCGGGAGACCCCCGCGTGGGTCGAGCGCGCTGAGTGTCCCGTGCCTCTTTCCTAGGCAAAGAGAAGGAGCCCCTGGAGTCGCAGTACCAGGTGGGCCCGCTGTTGGGCAGCGGTGGCTTCGGCTCGGTCTACTCTGGCATCCGCGTCGCCGACAACTTGCCGGTGAGTGGGCAACGCCGCCTCGGGGAGGGCGCGCGGAGGGATGCTTTGAGCAGGAGGGTCTGATGGAGACTCGGGGGTCTTCTTCCAGGTGGCCATTAAGCACGTGGAGAAGGACCGGATTTCCGATTGGGGAGAACTGGTGAGTGAGCCGGAGCCAGAGACCCTCTCATGGGTGGGTGGGTGCGACGGGCGTGCCGGAACCGGCGCGCTAACCCTCGCCCTCACTCTGCAGCCCAATGGCACCCGAGTGCCCATGGAAGTGGTCCTGTTGAAGAAGGTGCGCTCAAAAAGCCTGAACTCACCGCGACGTCTGTCGAGAAGTTTCTGATCGAAAAGTTCGACAGCGTCTCCGACCTGATGCAGCTCTCGGAGGGCGAAGAATCTCGTGCTTTCAGCTTCGATGTAGGAGGGCGTGGATATGTCCTGCGGGTAAATAGCTGCGCCGATGGTTTCTACAAAGATCGTTATGTTTATCGGCACTTTGCATCGGCCGCGCTCCCGATTCCGGAAGTGCTTGACATTGGGGAATTCAGCGAGAGCCTGACCTATTGCATCTCCCGCCGTGCACAGGGTGTCACGTTGCAAGACCTGCCTGAAACCGAACTGCCCGCTGTTCTGCAGCCGGTCGCGGAGGCCATGGATGCGATCGCTGCGGCCGATCTTAGCCAGACGAGCGGGTTCGGCCCATTCGGACCGCAAGGAATCGGTCAATACACTACATGGCGTGATTTCATATGCGCGATTGCTGATCCCCATGTGTATCACTGGCAAACTGTGATGGACGACACCGTCAGTGCGTCCGTCGCGCAGGCTCTCGATGAGCTGATGCTTTGGGCCGAGGACTGCCCCGAAGTCCGGCACCTCGTGCACGCGGATTTCGGCTCCAACAATGTCCTGACGGACAATGGCCGCATAACAGCGGTCATTGACTGGAGCGAGGCGATGTTCGGGGATTCCCAATACGAGGTCGCCAACATCTTCTTCTGGAGGCCGTGGTTGGCTTGTATGGAGCAGCAGACGCGCTACTTCGAGCGGAGGCATCCGGAGCTTGCAGGATCGCCGCGGCTCCGGGCGTATATGCTCCGCATTGGTCTTGACCAACTCTATCAGAGCTTGGTTGACGGCAATTTCGATGATGCAGCTTGGGCGCAGGGTCGATGCGACGCAATCGTCCGATCCGGAGCCGGGACTGTCGGGCGTACACAAATCGCCCGCAGAAGCGCGGCCGTCTGGACCGATGGCTGTGTAGAAGTACTCGCCGATAGTGGAAACCGACGCCCCAGCACTCGTCCGAGGGCAAAGGAATAGTCGACTACACGGACTTTGATGGTGAGCCAGGCCCC
