# Supplementary material for: Defining the mutation signatures of DNA polymerase θ in cancer genomes
Source: NAR Cancer. 2020 Aug 27;2(3):zcaa017. doi: 10.1093/narcan/zcaa017 (PMC7454005; doi:10.1093/narcan/zcaa017)
Supplement: zcaa017_Supplemental_Files [file zcaa017_supplemental_files.zip › Supplementary Table 2_Coverage and cut off number for the proximal end-joining NGS data.docx]

**Supplementary Table2.** Coverage and cut off number for the proximal end-joining NGS data

**Experiment 1**

| **Sample name** | **Mapped count** | **Input sequence number** | **Coverage** | **Poisson distribution (99% confidence)** | **Poisson distribution (95% confidence)** | **Poisson distribution (90% confidence)** | **Cutoff** |
| --- | --- | --- | --- | --- | --- | --- | --- |
| DR-U2OS-sh53BP1-1 | 418004 | 6000 | 69.67 | 50.51 | 55.74 | 58.59 | 50 |
| DR-U2OS-shcontrol-1 | 360304 | 6000 | 60.05 | 42.29 | 47.1 | 49.74 | 42 |
| DR-U2OS-shDNA-PKcs | 186508 | 6000 | 31.08 | 18.4 | 21.72 | 23.56 | 18 |
| F10-sh53BP1-1 | 37184 | 6000 | 6.2 | 0.77 | 1.93 | 2.64 | 1 |
| F10-shcontrol-1 | 123861 | 6000 | 20.64 | 10.37 | 12.98 | 14.44 | 10 |
| F10-shDNA-PKcs-1 | 13507 | 3000 | 4.5 | ND | 0.85 | 1.42 | 1 |
| G6-sh53BP1-1 | 500628 | 6000 | 83.44 | 62.45 | 68.21 | 71.35 | 62 |
| G6-shcontrol-1 | 338713 | 6000 | 56.45 | 39.24 | 43.89 | 46.44 | 39 |
| G6-shDNA-PKcs-1 | 68886 | 3000 | 22.96 | 12.11 | 14.89 | 16.44 | 12 |

**Experiment 2**

| **Sample name** | **Mapped count** | **Input sequence number** | **Coverage** | **Poisson distribution (99% confidence)** | **Poisson distribution (95% confidence)** | **Poisson distribution (90% confidence)** | **Cutoff** |
| --- | --- | --- | --- | --- | --- | --- | --- |
| DR-U2OS-sh53BP1-2 | 113282 | 6000 | 18.88 | 9.07 | 11.54 | 12.93 | 9 |
| DR-U2OS-shcontrol-2 | 130732 | 6000 | 21.79 | 11.22 | 13.92 | 15.43 | 11 |
| DR-U2OS-shDNA-PKcsi-2 | 377973 | 6000 | 63 | 44.8 | 49.74 | 52.44 | 44 |
| F10-sh53BP1-2 | 63485 | 6000 | 10.58 | 3.34 | 5.05 | 6.04 | 3 |
| F10-shcontrol-2 | 448546 | 6000 | 74.76 | 54.91 | 60.33 | 63.29 | 54 |
| F10-shDNA-PKcs-2 | 107973 | 6000 | 18 | 8.43 | 10.83 | 12.18 | 8 |
| G6-sh53BP1-2 | 406738 | 6000 | 67.79 | 48.9 | 54.04 | 56.85 | 48 |
| G6-shcontrol-2 | 320790 | 6000 | 53.47 | 36.73 | 41.24 | 43.71 | 36 |
| G6-shDNA-PKcs-2 | 310128 | 6000 | 51.69 | 35.23 | 39.66 | 42.09 | 35 |

**Experiment 3**

| **Sample name** | **Mapped count** | **Input sequence number** | **Coverage** | **Poisson distribution (99% confidence)** | **Poisson distribution (95% confidence)** | **Poisson distribution (90% confidence)** | **Cutoff** |
| --- | --- | --- | --- | --- | --- | --- | --- |
| DR-U2OS-sh53BP1-3 | 63465 | 6000 | 10.58 | 3.34 | 5.05 | 6.04 | 3 |
| DR-U2OS-shcontrol-3 | 292495 | 6000 | 48.75 | 32.78 | 37.06 | 39.42 | 32 |
| DR-U2OS-shDNA-PKcs-3 | 367888 | 6000 | 61.31 | 43.37 | 48.23 | 50.9 | 43 |
| F10-sh53BP1-3 | 43395 | 6000 | 7.23 | 1.33 | 2.64 | 3.42 | 1 |
| F10-shcontrol-3 | 291067 | 6000 | 48.51 | 32.58 | 36.86 | 39.2 | 32 |
| F10-shDNA-PKcs-3 | 84146 | 6000 | 14.02 | 5.62 | 7.68 | 8.85 | 5 |
| G6-sh53BP1-3 | 415476 | 6000 | 69.25 | 50.15 | 55.36 | 58.2 | 50 |
| G6-shcontrol-3 | 130254 | 6000 | 21.71 | 11.16 | 13.85 | 15.36 | 11 |
| G6-shDNA-PKcs-3 | 141188 | 6000 | 23.53 | 12.54 | 15.36 | 16.94 | 12 |
