# Supplementary material for: Defining the mutation signatures of DNA polymerase θ in cancer genomes
Source: NAR Cancer. 2020 Aug 27;2(3):zcaa017. doi: 10.1093/narcan/zcaa017 (PMC7454005; doi:10.1093/narcan/zcaa017)
Supplement: zcaa017_Supplemental_Files [file zcaa017_supplemental_files.zip › Supplementary Table 5_Coverage and cut off number for the distal end-joining NGS data.docx]

**Supplementary Table 5.** Coverage and cut off number for the distal end-joining NGS data

| **Sample name** | **Mapped count** | **Input sequence number** | **Coverage** | **Poisson distribution (99% confidence)** | **Cutoff** |
| --- | --- | --- | --- | --- | --- |
| 7F2-WT-1 | 1037670 | 7930 | 130.85 | 104.51 | 104 |
| 7F2-WT-2 | 1194458 | 7930 | 150.63 | 122.34 | 122 |
| 7F2-WT-3 | 911417 | 7930 | 114.93 | 90.26 | 90 |
| 7F2-F7-1 | 1098630 | 7930 | 138.54 | 111.42 | 111 |
| 7F2-F7-2 | 1128046 | 7930 | 142.25 | 114.77 | 114 |
| 7F2-F7-3 | 1178763 | 7930 | 148.65 | 120.54 | 120 |
| 7F2-F10-1 | 1031397 | 7930 | 130.06 | 103.80 | 103 |
| 7F2-F10-2 | 1170860 | 7930 | 147.65 | 119.64 | 119 |
| 7F2-F10-3 | 809145 | 7930 | 102.04 | 78.80 | 78 |
