# Supplementary material for: Defining the mutation signatures of DNA polymerase θ in cancer genomes
Source: NAR Cancer. 2020 Aug 27;2(3):zcaa017. doi: 10.1093/narcan/zcaa017 (PMC7454005; doi:10.1093/narcan/zcaa017)
Supplement: zcaa017_Supplemental_Files [file zcaa017_supplemental_files.zip › Supplementary_Figs.pdf]

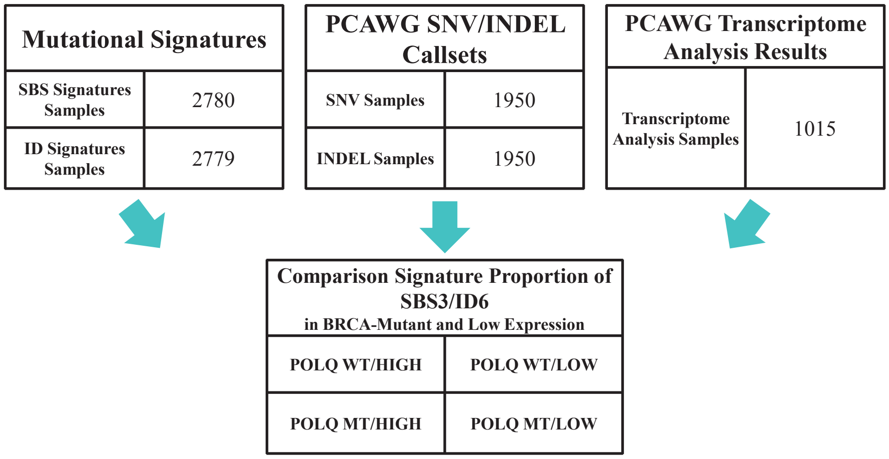

Supplementary Fig. 1

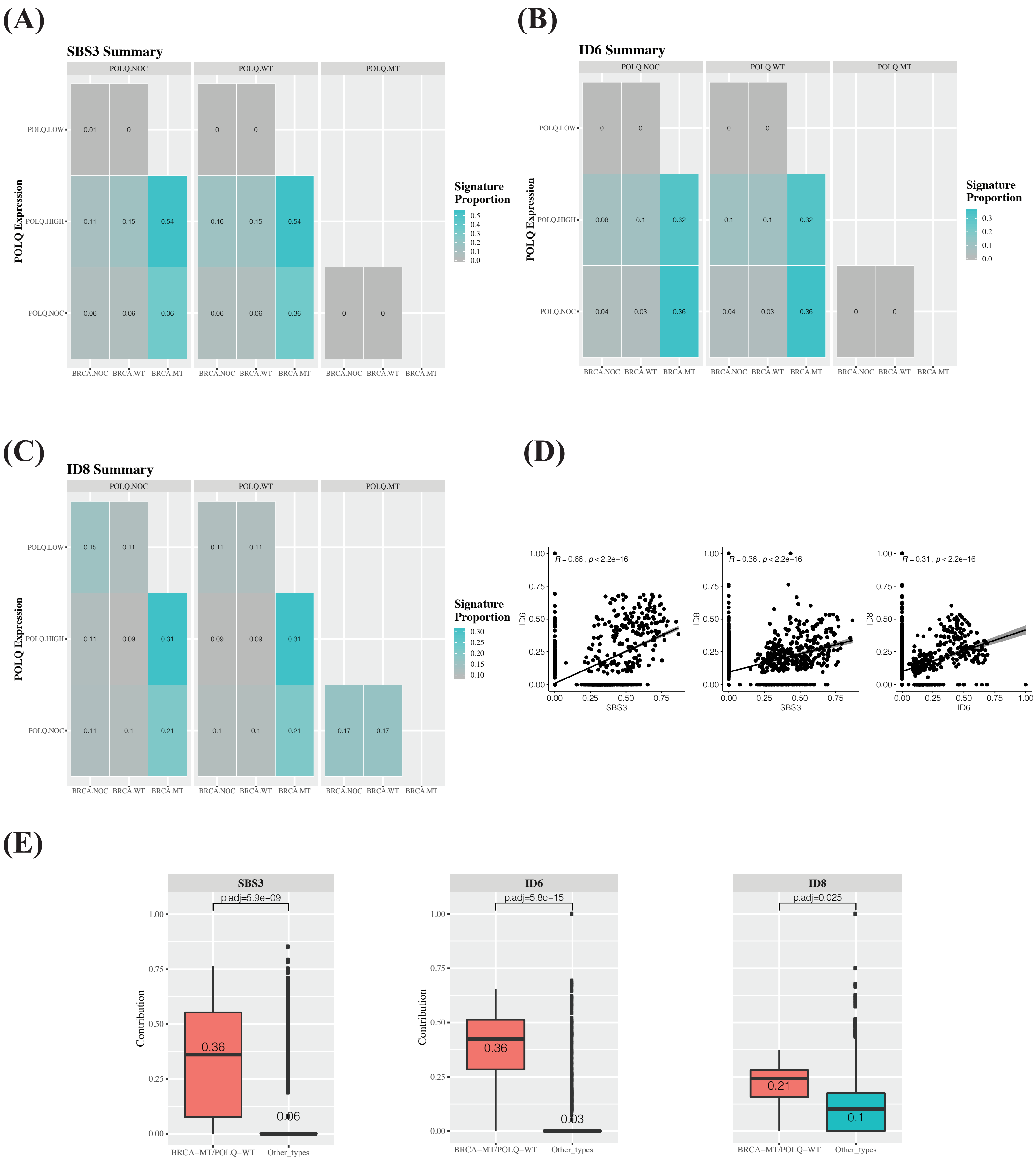

Supplementary Fig. 2

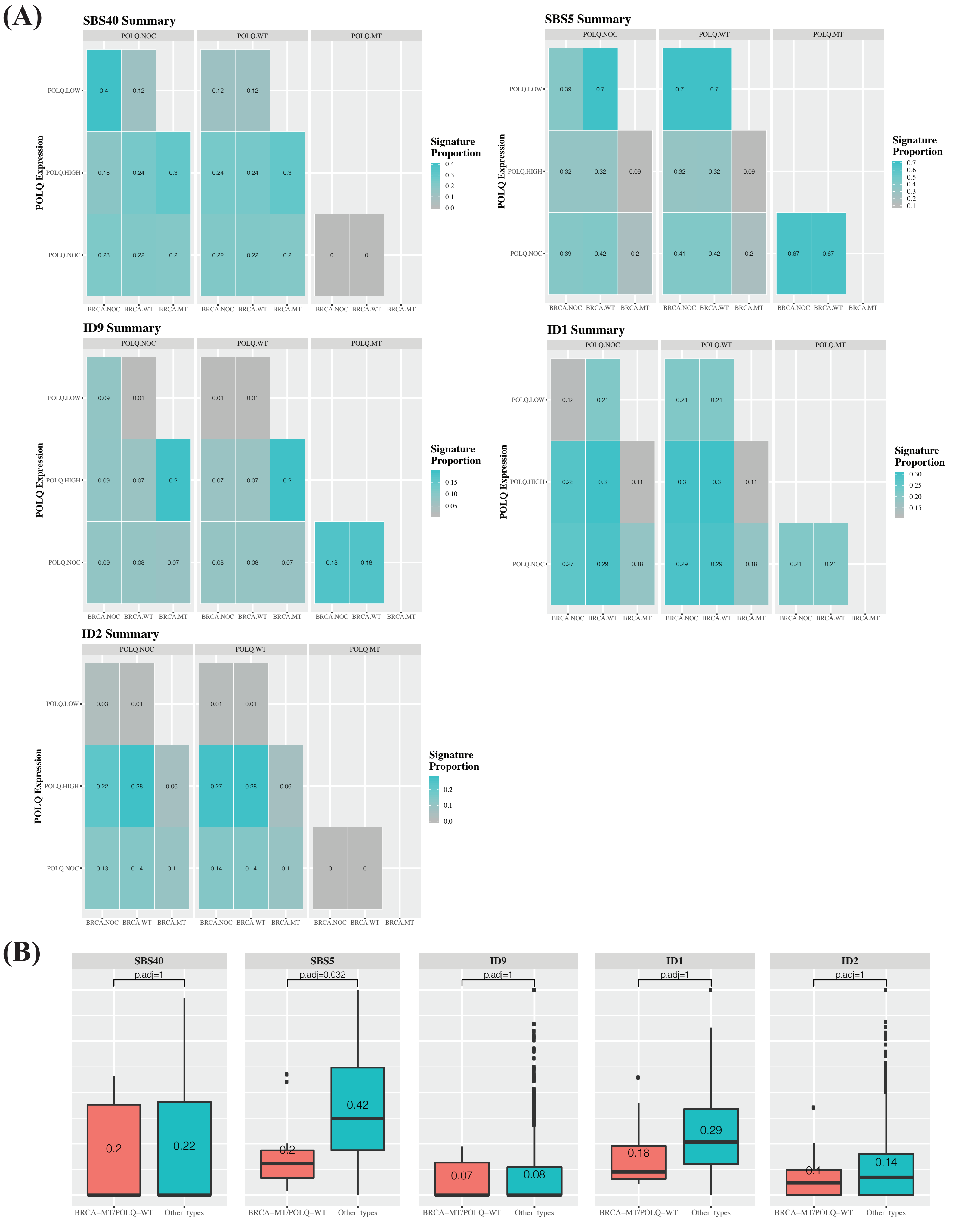

Supplementary Fig. 3

(A)

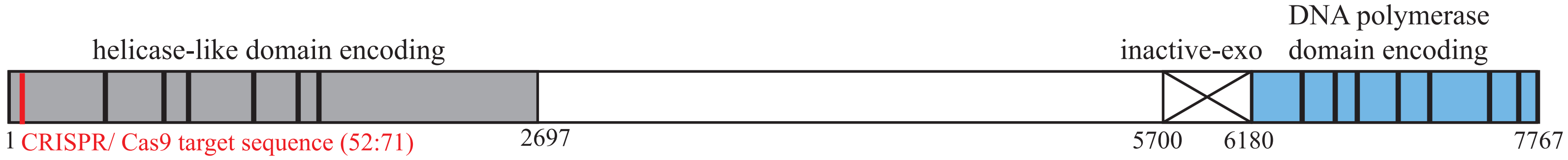

(B)

wild-type allele
1-**MNLLRRSGKRRRSESGSDSFSGSGGDSSASPQFLSGSVLSPPPGLGRCLKAAAAGECKPTVPDYEIDKLLLANWGLPKAVLEKYHSFGVKKMFEWQAECLLLGQVLEGKNLVYSAPTSAG**-120

DRU2OS F5 2 nt deletion allele
1-**MNLLRRSGKRRRSESGSDSFSG**RR*
DRU2OS F10 463 nt insertion allele
1-**MNLLRRSGKRRRSESGSDSFSGS**TPPIDVTMGTYVIIDVNGRGSLGGQPGGPFTVSYVTRNSIYGL*
DRU2OS G6 23 nt insertion allele
1-**MNLLRRSGKRRRSESGSDSFSGS**DSFSGSVSAVTAVPAPSSSPGPC*

7F2 F7 1 nt deletion allele
1-**MNLLRRSGKRRRSESGSDSFSG**RAVTAVPAPSSSPGPC*
7F2 F7 530 nt insertion allele
1-**MNLLRRSGKRRRSESGSDSFSGSG**DPTSLAPHERLTDLAVVPREKPHTGAAARELVCCINNEIKLMTFQFVHTQRLNTASVRKQFSQLFRAENYKKNKKXSVRFAQQHRSQKEKEGGHSRRACGRQKVQHRRDERSEA*
7F2 F10 2 nt deletion allele
1-**MNLLRRSGKRRRSESGSDSFSG**RR*
7F2 F10 10 nt deletion allele
1-**MNLLRRSGKRRRSESGSDSFSG**TAVPAPSSSPGPC*

(C)

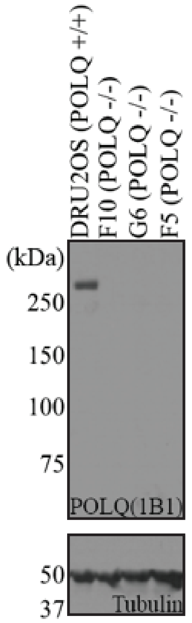

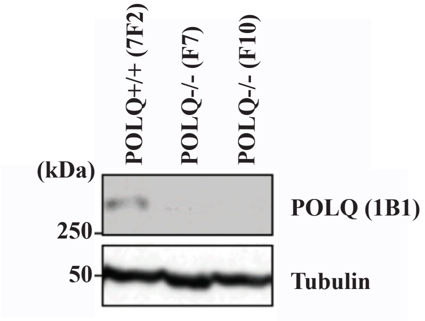

Supplementary Fig. 4

Wild-type allele
ctggcgggaagatgtccgcagctgttgccaggccagggttctcccgagagggaggacgctgg
gactgtggcttgccctgatcggccgagaagagtttgccATGAATCTTCTGCGTCGGAGTGGG
AAACGGCGGCGTTCAGAATCAGGCTCAGATTCGTTCTCGGGAAGCGGCGGTGACAGCAGTGC
CAGCCCCCAGTTCCTCTCCGGGTCCGTGCTGAGCCCGCCGCCCGGCCTTGGTCGCTGCCTGA
AGGCCGCAGCTGCAG

DRU2OS F5 allele
ctggcgggaagatgtccgcagctgttgccaggccagggttctcccgagagggaggacgctgg
gactgtggcttgccctgatcggccgagaagagtttgccATGAATCTTCTGCGTCGGAGTGGG
AAACGGCGGCGTTCAGAATCAGGCTCAGATTCGTTCTCGGGA**CGGCGGTGACAGCAGTGC
CAGCCCCCAGTTCCTCTCCGGGTCCGTGCTGAGCCCGCCGCCCGGCCTTGGTCGCTGCCTGA
AGGCCGCAGCTGCAG

DRU2OS F10 allele
ctggcgggaagatgtccgcagctgttgccaggccagggttctcccgagagggaggacgctgg
gactgtggcttgccctgatcggccgagaagagtttgccATGAATCTTCTGCGTCGGAGTGGG
AAACGGCGGCGTTCAGAATCAGGCTCAGATTCGTTCTCGGGAAG**TACGCCCCCTATTGACGT**
TACTATGGGAACATACGTCATTATTGACGTCAATGGGCGGGGGTCGTTGGGCGGTCAGCCAG
GCGGGCCATTTACCGTAAGTTATGTAACGCGGAACTCCATATATGGGCTATGAACTAATGAC
CCCGTAATTGATTACTATTAATAACTAGTCAATAATCAATGTCAACGCGTATATCTGGCCCG
TACATCGCGAAGCAGCGCAAAACGCCTAACCCTAAGCAGATTCTTCATGCAATTGTCGGTCA
AGCCTTGCCTTGTTGTAGCTTAAATTTTGCTCGCGCACTACTCAGCGACCTCCAACACACAA
GCAGGGAGCAGATACTGGCTTAACTATGCGGCATCAGAGCAGATTGTACTGAGAGTGCACCA
TAGGGGATCGGGAGATCTCCCGATCCGTCGACGTCAGGTGGCACTTTTCGGGGAAATGTGCG
CGGATACATATCGGCGGTGACAGCAGTGCCAGCCCCCAGTTCCTCTCCGGGTCCGTGCTGAG
CCCGCCGCCCGGCCTTGGTCGCTGCCTGAAGGCCGCAGCTGCAG

DRU2OS G6 allele
ctggcgggaagatgtccgcagctgttgccaggccagggttctcccgagagggaggacgctgg
gactgtggcttgccctgatcggccgagaagagtttgccATGAATCTTCTGCGTCGGAGTGGG
AAACGGCGGCGTTCAGAATCAGGCTCAGATTCGTTCTCGGGAAG**TGATTCGTTCTCGGGAAG**
TGTCTCGGCGGTGACAGCAGTGCCAGCCCCCAGTTCCTCTCCGGGTCCGTGCTGAGCCCGCC
GCCCGGCCTTGGTCGCTGCCTGAAGGCCGCAGCTGCAG

EDS-7F2 F7 allele 1
ctggcgggaagatgtccgcagctgttgccaggccagggttctcccgagagggaggacgctgg
gactgtggcttgccctgatcggccgagaagagtttgccATGAATCTTCTGCGTCGGAGTGGG
AAACGGCGGCGTTCAGAATCAGGCTCAGATTCGTTCTCGGGAAG*GGCGGTGACAGCAGTGC
CAGCCCCCAGTTCCTCTCCGGGTCCGTGCTGAGCCCGCCGCCCGGCCTTGGTCGCTGCCTGA
AGGCCGCAGCTGCAG

EDS-7F2 F7 allele 2
ctggcgggaagatgtccgcagctgttgccaggccagggttctcccgagagggaggacgctgg
gactgtggcttgccctgatcggccgagaagagtttgccATGAATCTTCTGCGTCGGAGTGGG
AAACGGCGGCGTTCAGAATCAGGCTCAGATTCGTTCTCGGGAAG**TGGAGACCCGACTTCCCT**
GGCGCCCCACGAGAGGCTCACTGACCTCGCCGTCGTACCTCGTGAGAAACCCCACACTGGGG
CCGCCGCTCGAGAACTTGTATGTTGCATTAATAATGAGATCAAACTCATGACCTTCCAGTTC
GTCCATACTCAACGCCTGAATACTGCCAGTGTGCGCAAACAATTTAGCCAACTCTTCCGCGC
GGAAAACTATAAGAAAAATAAAAAACRAAGTGTAAGRTTTGCACAACAGCACCGCTCCCAAA
AAGAAAAGGAAGGTGGGCATTCACGGCGTGCCTGCGGCCGACAAAAAGTACAGCATCGGCGA

GATGAGCGCTCAGAAGCTTAGAAAGATTATATAAAGTTTCCAAGGCCATTGGACAAAACGTG
GTCCACTGGAGAAGGGAATGGCAAACCACTTCAATAGTCTTGCCTTGAGAACCCCATGAACA
GTATCAAAGTGATCATTTTAAAAGAAAAATTTATCAGTCATTTCTTATTTTAAATTCTTAGT
TGGCTTTCAGTGACTTCGGCGGTGACAGCAGTGCCAGCCCCCAGTTCCTCTCCGGGTCCGTG
CTGAGCCCGCCGCCCGGCCTTGGTCGCTGCCTGAAGGCCGCAGCTGCAG

EDS-7F2 F10 allele 1
ctggcgggaagatgtccgcagctgttgccaggccagggttctcccgagagggaggacgctgg
gactgtggcttgccctgatcggccgagaagagtttgccATGAATCTTCTGCGTCGGAGTGGG
AAACGGCGGCGTTCAGAATCAGGCTCAGATTCGTTCTCGGGAAG**GCGGTGACAGCAGTGC
CAGCCCCCAGTTCCTCTCCGGGTCCGTGCTGAGCCCGCCGCCCGGCCTTGGTCGCTGCCTGA
AGGCCGCAGCTGCAG

EDS-7F2 F10 allele 2
ctggcgggaagatgtccgcagctgttgccaggccagggttctcccgagagggaggacgctgg
gactgtggcttgccctgatcggccgagaagagtttgccATGAATCTTCTGCGTCGGAGTGGG
AAACGGCGGCGTTCAGAATCAGGCTCAGATTCGTTCTCGGGAA**********CAGCAGTGC
CAGCCCCCAGTTCCTCTCCGGGTCCGTGCTGAGCCCGCCGCCCGGCCTTGGTCGCTGCCTGA
AGGCCGCAGCTGCAG

Supplementary Fig. 4D

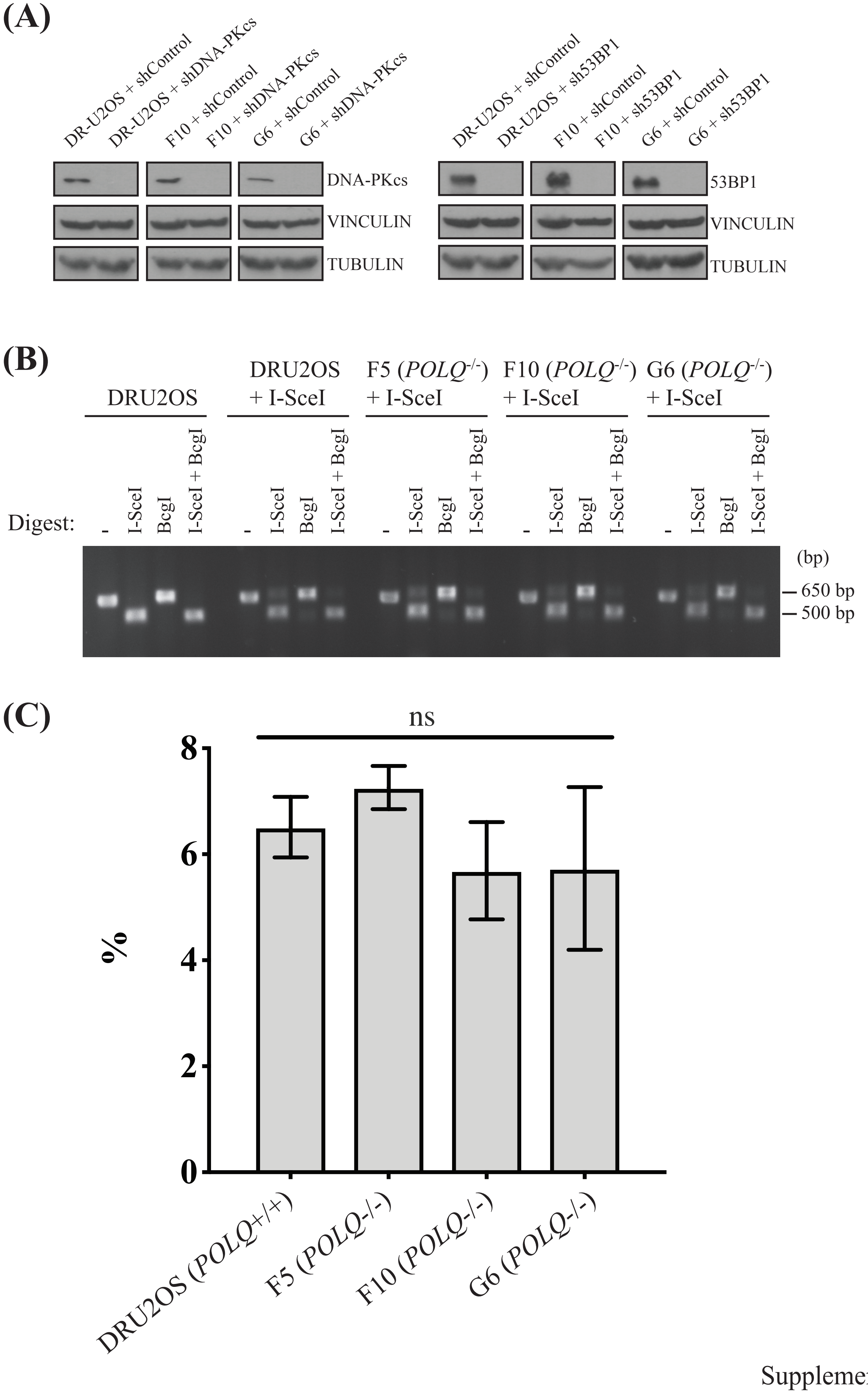

Supplementary Fig. 5

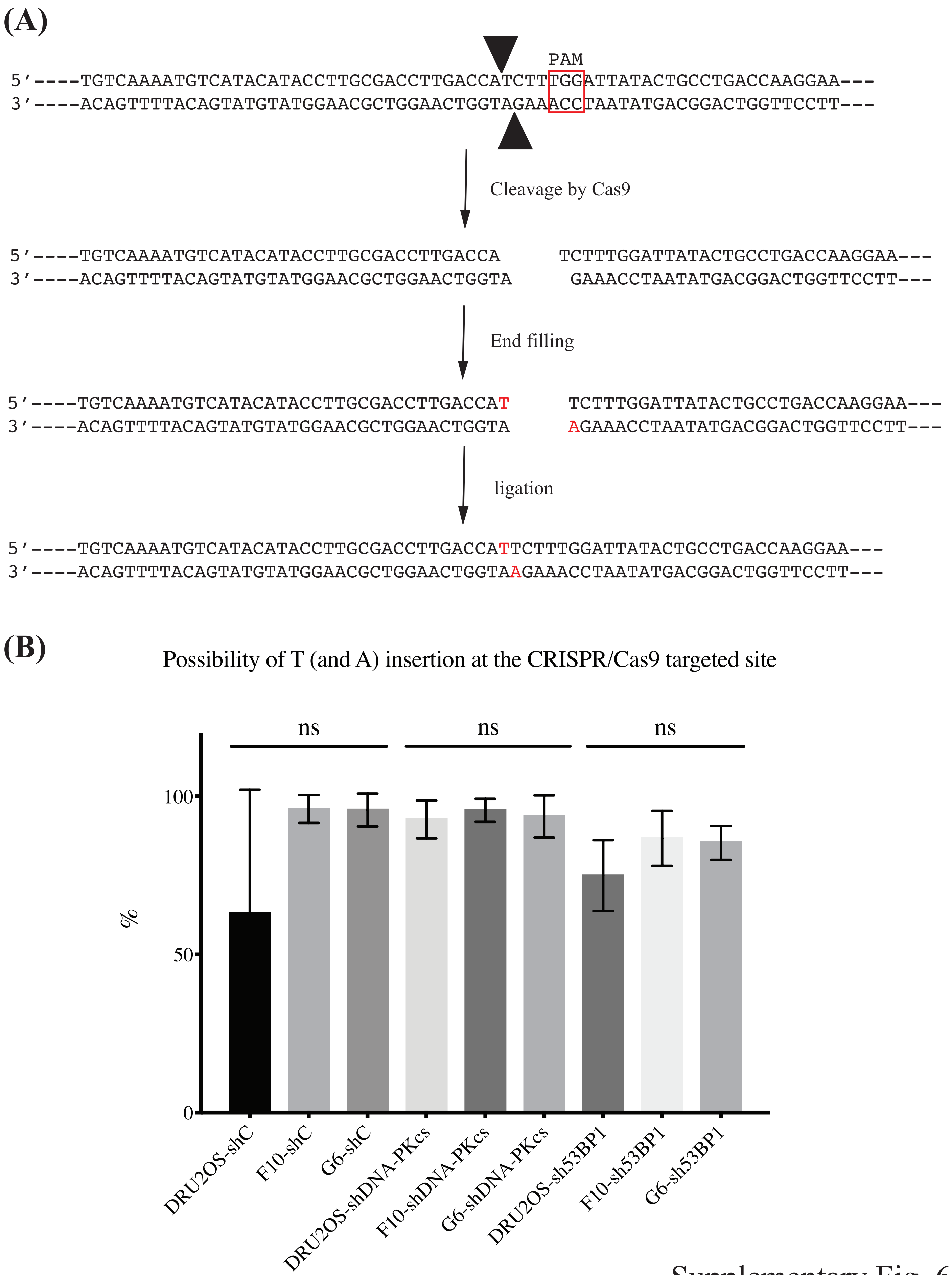

Supplementary Fig. 6
